# Critical Roles of Balanced T Helper 9 Cells and Regulatory T Cells in Allergic Airway Inflammation and Tumor Immunity

**DOI:** 10.1155/2021/8816055

**Published:** 2021-03-01

**Authors:** Muhua Huang, Jingcheng Dong

**Affiliations:** ^1^Department of Integrative Medicine, Huashan Hospital, Fudan University, Shanghai 200040, China; ^2^Institute of Integrative Medicine, Fudan University, Shanghai 200040, China

## Abstract

CD4^+^T helper (Th) cells are important mediators of immune responses in asthma and cancer. When counteracted by different classes of pathogens, naïve CD4^+^T cells undergo programmed differentiation into distinct types of Th cells. Th cells orchestrate antigen-specific immune responses upon their clonal T-cell receptor (TCR) interaction with the appropriate peptide antigen presented on MHC class II molecules expressed by antigen-presenting cells (APCs). T helper 9 (Th9) cells and regulatory T (Treg) cells and their corresponding cytokines have critical roles in tumor and allergic immunity. In the context of asthma and cancer, the dynamic internal microenvironment, along with chronic inflammatory stimuli, influences development, differentiation, and function of Th9 cells and Treg cells. Furthermore, the dysregulation of the balance between Th9 cells and Treg cells might trigger aberrant immune responses, resulting in development and exacerbation of asthma and cancer. In this review, the development, differentiation, and function of Th9 cells and Treg cells, which are synergistically regulated by various factors including cytokine signals, transcriptional factors (TFs), costimulatory signals, microenvironment cues, metabolic pathways, and different signal pathways, will be discussed. In addition, we focus on the recent progress that has helped to achieve a better understanding of the roles of Th9 cells and Treg cells in allergic airway inflammation and tumor immunity. We also discuss how various factors moderate their responses in asthma and cancer. Finally, we summarize the recent findings regarding potential mechanisms for regulating the balance between Th9 and Treg cells in asthma and cancer. These advances provide opportunities for novel therapeutic strategies that are aimed at reestablishing the balance of these cells in the diseases.

## 1. Introduction

When detected of a wide variety of pathogens, the adaptive immune system utilizes T lymphocytes to establish and maintain immune response [1, 2]. Upon interaction with antigen presented by antigen-presenting cells (APCs) such as dendritic cells (DCs), naïve CD4^+^T cells can differentiate into distinct types of CD4^+^T helper cells (Th cells) including Th1, Th2, Th17, Th9, Th22, follicular T helper (Tfh), and partial regulatory T cell (Treg) subsets [3]. The differentiation process is governed predominantly by microenvironmental cues such as cytokines signals, costimulatory signals, inflammatory milieu, and to some extent, the strength of the interaction of the T-cell antigen receptor (TCR) with antigen [4]. Importantly, a balanced state of Th cell populations is required for triggering an effective inflammatory response and for remaining being immune-tolerant homeostasis and, thus, for attenuating the exuberant immune response in disease conditions [1].

Th9 cells which exhibit a strong proinflammatory activity mediate allergic inflammation and tumor immunity [5]. The pathogenic function of Th9 cells is limited by Treg cells which suppress aberrant immune responses [2, 6, 7]. In addition, Treg cells play indispensable roles in preventing immune pathology induced by pathogens and in maintaining tolerance to allergens by regulating allergen-triggered immune response [8, 9]. Treg cells can also suppress antitumor immune response [10]. Recent insights into molecular and cellular mechanisms of asthma and cancer have indicated that Th9 cells and Treg cells acted in an opposing manner to regulate allergic and tumor-specific immune responses [11–14]. Meanwhile, several experiments have demonstrated that the imbalanced status between Th9 cells and Treg cells was closely associated with the pathogenesis of asthma and cancer [11, 15]. Despite the growing awareness regarding the importance of Th9 cells and Treg cells in regulating allergic airway inflammation and tumor immunity, the mechanisms underpinning the imbalance between these cells in experimental models of allergic airway inflammation or tumor and in asthma or cancer patients have not been thoroughly examined. There is evidence that multiple factors including cytokine signals, transcriptional factors (TFs), epigenetic regulators, microenvironment cues, metabolic pathways, and different signaling pathways synergistically regulate reciprocal development pathways and activation of Th9 cells and Treg cells [11, 16–20]. Th9 cells and Treg cells exhibit some degree of plasticity of coexpressing specific cytokines [19]. These concepts may be at the core of the mechanisms involved in regulating balance between these cells in asthma and cancer (discussed in detail below). In this review, we describe recent studies exploring the roles of Th9 cells and Treg cells in allergic airway inflammation and cancer. Moreover, we discuss how different factors such as cytokine signals, TFs, metabolic pathways, and different signaling pathways regulate the development, function, plasticity, and balance of these cells. Specifically, we focus on potential methods and mechanisms of reestablishing the balance between Th9 and Treg cells that control the development of asthma and cancer.

## 2. Characterization of the Cell Subsets

### 2.1. Th9 Cells

Almost three decades before the first identification of Th9 cells in vivo, it was reported that production of interleukin-9 (IL-9) by CD4^+^T cells was dependent on IL-2, induced by IL-4 and transforming growth factor-*β* (TGF-*β*) signaling, and further enhanced by IL-1 and IL-25, while IFN-*γ* was considered as an inhibitory cytokine of IL-9 generation [21–23]. Studies using *Trichuris muris*-infected mice where TGF-*β* signaling is blocked in CD4^+^T cells and *Nippostrongylus brasiliensis*-infected IL-9 fluorescent reporter mice defined these IL-9-producing CD4^+^T cells as Th9 cells and demonstrated that Th9 cells could expulse the parasitic worm [24, 25]. Subsequent experiments further demonstrated that TGF-*β* signaling and additional stimuli such as calcitonin gene-related peptide (CGRP), TNF ligand-related molecule 1A (TL1A), and thymic stromal lymphopoietin (TSLP) were required for the development of Th9 cells [26–29]. Meanwhile, extensive research indicated that Th9 cells contributed to allergic pathologies, development of brain inflammation of experimental autoimmune encephalomyelitis (EAE), and colitis [13, 30–33]. Th9 cells also play a major role in antitumor immunity [34, 35]. It seems likely that Th9 cells utilize multiple mechanisms for promoting allergic inflammation and restraining tumor growth. Th9 cells interact with multiple cell types including mast cells (MCs), innate lymphoid cells (ILCs), and dendritic cells (DCs) to facilitate coordinated regulation of allergic airway inflammation and tumor development [36–38]. IL-9 has been considered a predominant signature cytokine of Th9 cells and plays critical roles in the allergic and antitumor immune responses mediated by Th9 cells [38, 39]. Furthermore, mouse and human Th9 cells also secret other cytokines such as IL-21, IL-10, IL-17, and IL-22, which orchestrate the immune responses in cancer and allergic asthma [30, 40–42].

### 2.2. Cytokines, Transcription Factors (TFs), and Signal Transduction Pathways for Development and Differentiation of Th9 Cells

**Figure 1:**
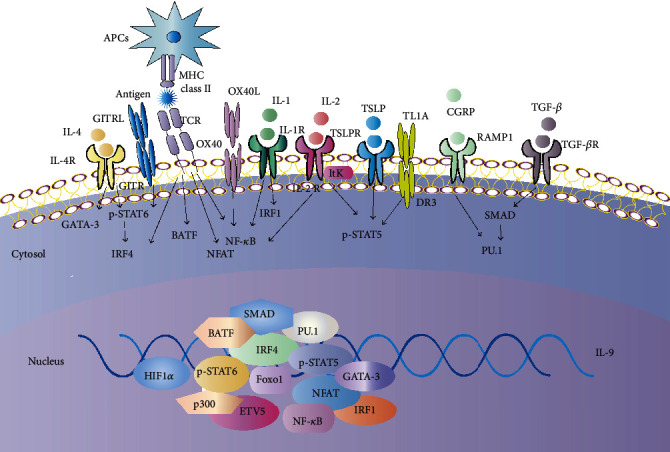
Cytokines, transcription factors (TFs), and signal transduction pathways for interleukin-9 (IL-9) production and Th9 cell differentiation. The development and differentiation of Th9 cell mainly require TFs including IFN-regulatory factor (IRF4), PU.1, signal transducer and activator of transcription 6 (STAT6)，STAT5, and drosophila mothers against decapentaplegic protein (SMAD) which function downstream of cytokine signals including IL-4, IL-2, and transforming growth factor-*β* (TGF-*β*) signals. T cell receptor (TCR) and TCR-dependent signals such as nuclear factor of activated T cells (NFAT) and costimulatory signals including glucocorticoid-induced TNFR-related protein (GITR) and OX40 signals also have important roles in IL-9 transcription upon interaction with antigen-presenting cells (APCs). Accessory cytokines such as IL-1, IL-2, and costimulatory molecule OX40 induce nuclear factor-*κ*B (NF-*κ*B) signaling to drive IL-9 transcription and generation of Th9 cell. In addition, downstream signals of the TCR leading to the expression of adaptor-related protein complex 1 (AP-1) family member basic leucine zipper transcription factor ATF-like- (BATF-) JUN may contribute to IL-9 expression in a cooperative manner with IRF4. Essentially, these TFs contribute to chromatin modifications at the *Il9* locus and the initiation of IL-9 expression. Coactivator histone acetyltransferase p300 promotes accessibility of the *Il9* locus. Other factors including forkhead box-O1 (Foxo1), ETV5, GATA-binding protein 3 (GATA-3), IRF1, hypoxia-inducible factor 1a (HIF1*α*), and IL-2-inducible tyrosine kinase (Itk), as well as additional stimuli including calcitonin gene-related peptide (CGRP), TNF ligand-related molecule 1A (TL1A), and thymic stromal lymphopoietin (TSLP) further enhance IL-9 production by Th9 cell.

Induction or activation of specific TFs in response to TCR signaling, cytokines, and other molecules such as costimulatory molecules determines development and differentiation programs of Th cells [43]. In contrast to other T helper cell subsets, a series of TFs constitute a regulatory network to control the fate commitment of Th9 cells and a single “master” TF has yet been identified [5]. Major Th9 regulatory TFs include IFN-regulatory factor (IRF4), PU.1, ETV5, GATA-binding protein 3 (GATA-3), drosophila mothers against decapentaplegic protein (SMAD) 2/3/4, IRF1, basic leucine zipper transcription factor ATF-like (BATF), nuclear factor-*κ*B (NF-*κ*B) RelB/p52, signal transducer and activator of transcription 6 (STAT6), and STAT5 [5, 11, 20, 34, 44, 45]. These TFs function downstream of various signals including IL-4, IL-2, TGF-*β*1, and IL-1 signals; TCR-dependent signals such as nuclear factor of activated T cell (NFAT) signal; costimulatory signals such as tumor necrosis factor receptor (TNFR) family members, glucocorticoid-induced TNFR-related protein (GITR) and OX40 (Tnfrsf4), and IL-2 inducible tyrosine kinase (Itk); and TL1A signals in the induction of IL-9 and development of Th9 lineage [11, 16, 46–50]. The induction and modulation of IL-9 protein expression require distinct cis-elements closed to the *Il9* gene locus including several evolutionally conserved noncoding sequences (CNSs) [51]. CNS1, CNS2, and CNS-6 bind to SMAD2/3/4, STAT5, STAT6, NFAT, and additional factors to regulate IL-9 production [52]. Interestingly, Th9 cell differentiation is controlled by competition between IL-2-STAT5 and IL-21-STAT3 pathways [53]. A recent study found that transcription factor forkhead box-O1 (Foxo1), a member of the Foxo family, could bind to and transactivate the *Il9* and *Irf4* promoters to control the development of Th9 cells [54] (Figure 1).

### 2.3. Treg Cells

The initial evidence of the existence of Treg cells came from research of organ-specific autoimmune disease [55, 56]. Adoptive transfer of particular CD4^+^T cells bearing particular molecular markers CD25 (the IL-2 receptor *α* chain) and forkhead box P3 (Foxp3) encoded by the X-chromosome into recipient thymectomy mice can inhibit damage of various organs caused by chronic inflammation and prevent the disease development [57]. Moreover, Treg cells are categorized into thymic Treg (tTreg) cells that arise in the thymus and peripherally induced Treg (pTreg) cells that differentiate in the periphery and in vitro-induced Treg (iTreg) cells [58]. Interestingly, both types of Treg cells are considered being interchangeable or reversible in regulating the immune response [59, 60]. Treg cells prevent inappropriate inflammatory responses and maintain immune homeostasis. They also have detrimental effects on tumor progression and infectious pathogen persistence [61]. Importantly, several researches suggested that their regulatory functions might depend on different modes of action including function modification or elimination of other immune cells like APCs [57]; inhibiting different aspects of effector Th cell effectiveness including suppression of the priming and expansion of aberrant Th cells [62]; blocking the migration of Th cells from the site of immunization [63]; inhibiting the differentiation of naïve precursors into pathogenic effector Th cells through exploiting a variety of TFs and cytokine signals [64]; utilizing cytotoxicity granzyme, perforin to inactivate responder T cells and delivering negative signals to effector Th cells [57, 65]; secretion of specific immunosuppressive cytokines such as IL-10, TGF-*β*, or IL-35 [57]; expression of cell surface inhibitory receptors such as cytotoxic T lymphocyte-associated protein 4 (CTLA-4) [66]; depletion of IL-2 by overexpression of the high-affinity IL-2R *α* chain [67]; and purine-mediated suppression by CD39-dependent conversion of ATP [68].

### 2.4. Cytokines, TFs, and Signal Transduction Pathways for Development and Differentiation of Treg Cells

**Figure 2:**
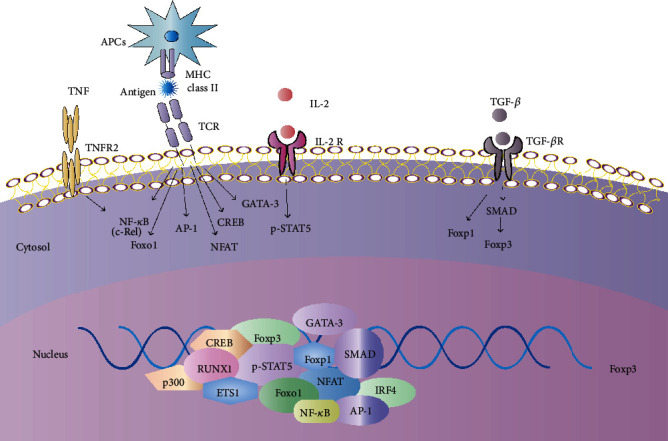
Cytokines, transcription factors (TFs), and signal transduction pathways for forkhead box P3 (Foxp3) induction and Treg cell differentiation. The development and differentiation of Treg cell mainly require TGF-*β*, IL-2 signals to activate Foxp3, and STAT5. The induction and maintenance of Foxp3 in Treg cell are regulated by distinct cis-elements within the *Foxp3* gene locus which are bound by SMAD2/3 and TFs such as STAT5, GATA-3, and cAMP response element-binding protein (CREB). The development and differentiation of Treg cell are also predominantly governed by TCR-dependent signals coupled with costimulation molecules such as TNFR2, which induce NF-*κ*B signaling to drive Foxp3 transcription. In addition, downstream signals of the TCR leading to the expression of AP-1 family member BATF-JUN may contribute to Foxp3 expression in a cooperative manner with IRF4. p300 affects the acetylation and function of multiple TFs such as Foxp3 in Treg cell. Other TFs including forkhead box P1 (Foxp1), NFAT, Foxo1, runt-related transcription factor (Runx1), and ETS1 further promote Foxp3 transcription and Treg cell function.

The development and differentiation of Treg cells require TGF-*β*, IL-2 signals to activate Foxp3, and STAT5 [69, 70]. Notably, proper STAT5 activation and the TGF-*β* signal play central roles in balanced development between Treg cells and Th9 cells [16, 71]. The development and differentiation of Treg cells are predominantly governed by TCR-dependent signals coupled with IL-2 receptor and costimulatory signals [72, 73]. Foxp3 can cooperate with a variety of TFs such as NFAT, Runx1 (also known as acute myeloid leukemia 1 (AML1)), Eos, BATF, Myb, IRF4, c-Maf, JunB, B lymphocyte-induced maturation protein 1 (Blimp1), BACH2, Foxo1, and different proteins including TIP6, AMBRA1, or histone deacetylase 7 (HDAC7) to regulate Treg cell lineage commitment and suppressive function [74, 75]. The induction and modulation of Foxp3 protein expression require distinct cis-elements within the *Foxp3* gene locus including its promoter and CNSs. CNS1, CNS2, and CNS3 bind to SMAD2/3, STAT5, GATA-3, and cAMP response element-binding protein (CREB) and additional factors to induce and regulate Foxp3 expression [76–80]. A recent study found that forkhead box-P1 (Foxp1), a sibling family member of Foxp3, could preserve a favorable state of chromatin modifications of the *Foxp3* locus by being physically associated with its promoter and enhancer region CNS2 in a TGF-*β*-dependent manner. Foxp1 sustains optimal expression of Foxp3 during the onset of extrathymic Treg cell induction [81] (Figure 2).

### 2.5. Reciprocal Developmental Pathways for the Generation of Th9 Cells and Treg Cells

**Figure 3:**
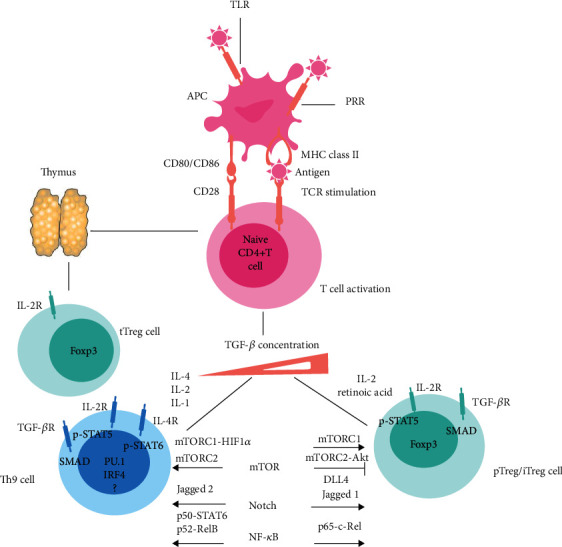
The reciprocal developmental pathways for the generation and differentiation of Th9 cell and Treg cell. The thymic Treg (tTreg) cell arises in the thymus. Transcription factor PU.1, IRF4, and Foxp3 are essential for differentiation of Th9 and Treg cell subsets, respectively. The local microenvironment strongly influences the processes that determine lineage differentiation of Th9 cell and Treg cell. Binding of conserved pathogen-derived molecules to pattern recognition receptors (PRRs) such as Toll-like receptors (TLRs), on the cell surface of APCs activates the production of various cytokines. Activated APCs present antigens to naïve CD4^+^T cell and promote differentiation of Th9 cell and Treg cell. The presence of high concentration of TGF-*β* in combination with IL-2 and retinoic acid (RA) is optimal for Foxp3 expression to facilitate periphery Treg (pTreg) cell or inducible Treg (iTreg) cell generation. By contrast, low concentration of TGF-*β* in combination with IL-4 and IL-2 induces differentiation of Th9 cell through induction of STAT6 and STAT5. In addition, IL-1*β* combined with IL-4 induces a Th9^IL-4+IL-1*β*^ cell subset. Different signaling pathways including the mechanistic target of rapamycin (mTOR), Notch, and NF-*κ*B signals are also required for the regulation of development and differentiation of Th9 cell and Treg cell.

Several studies have identified that a group of TFs including IRF4, BATF, NFAT, STAT5, SMAD2/3, and NF-*κ*B family of transcription factors, as well as the TGF-*β* signal, some costimulatory signals, and metabolic pathways, is shared between Th9 cells and Treg cells during their development and differentiation [20, 66, 82] (Figure 3).

#### 2.5.1. TGF-*β* Signal

Lying at the crossroads of the balance between Th9 cells and Treg cells, the TGF-*β* signal has a dual function in the development, differentiation, and their function [70, 83, 84]. In a different context, the TGF-*β* signal can induce either immunosuppressive Treg cells or proinflammatory Th9 cells depending on the amount of TGF-*β* and environmental factors such as cytokines presented to these cells [85, 86]. High concentration of TGF-*β* alone, or in combination with retinoic acid (RA) and IL-2, is optimal for Foxp3 expression [86]. TGF-*β* binding to its receptor leads to activation and recruitment of SMAD2 and SMAD3 into CNS1 within the *Foxp3* locus to promote differentiation of Treg cells [78, 87, 88]. TGF-*β* in combination with IL-4 in the presence of TCR and costimulatory signals induces development of the Th9 lineage [15, 19, 89]. TGF-*β* is also needed to induce expression of PU.1 by activating SMAD2 and SMAD4. SMAD2 and SMAD4 regulate IL-9 production through displacement of EZH2 and removal of suppressive H3K27 histone modification at the *Il9* locus during Th9 differentiation [20]. Activin-A, a TGF-*β* superfamily member, in combination with a low dose of TGF-*β* drives the generation of mouse Th9 cells in vitro [90]. Activin-A synergistically enhances TGF-*β*1-mediated Foxp3 expression leading to CD4^+^CD25^−^T cells conversion to Treg cells in vivo [91].

#### 2.5.2. STAT5

The transcription factor STAT5 functions downstream of several cytokines to regulate development of Th9 cells and Treg cells [53]. IL-2 signaling activates STAT5 in T cells and promotes Th9 cell development [53]. Recent studies found that activated STAT5 could bind to the promoter of the *Il9* locus and induce IL-9 transcription by reducing H3K9 histone methylation [53, 92]. A similar mechanism might be utilized by Treg cells. IL-2 signaling induces the activation of STAT5, which binds to and demethylates CNS2 to stabilize Foxp3 expression in Treg cells. On the other hand, IL-4 activates STAT6 to compete with STAT5 for the binding to similar sites in the *Foxp3* locus and to counteract STAT5 activity, thereby silencing Foxp3 transcription [76].

#### 2.5.3. IRF4

Interestingly, some TFs form a macromolecular complex to strengthen their role in regulating development and function of Th9 cells and Treg cells. Adaptor-related protein complex 1 (AP-1) family members BATF and JUN formed a trimeric complex with IRF4, referred to as activating protein-1 interferon regulatory factor (AP-1-IRF) composite elements (AICEs) [93]. AICEs regulate the differentiation of Th9 cells and Treg cells [50, 66, 94]. When naïve CD4^+^T cells were cultured under Th9 skewing conditions, IRF4 interacted with BATF and BATF3, binding to the *Il9* promoter to increase *Il9* gene transcription in Th9 cells [50, 95, 96]. AICEs also play a central role in the generation of effector Treg cells (eTreg) in peripheral organs. The study using genome-wide chromatin immunoprecipitation sequencing (ChIP-seq) analysis for *Junb*-deficient and *Junb*-sufficient Treg cells found that JunB colocalized with BATF and IRF4 at loci of some eTreg-related genes including costimulatory gene*-*inducible T cell costimulator (*Icos*) and coinhibitory gene *Ctla4*. JunB deficiency resulted in defect in the expression of ICOS and CTLA4 [66]. These data demonstrated that AICEs might be the critical regulator of differentiation and function of both Th9 cells and Treg cells (Table 1).

#### 2.5.4. The NF-*κ*B Signaling Pathway

The NF-*κ*B signaling pathway has important roles in the development and functional divergence of Th9 and Treg cells [97, 98]. Both canonical NF-*κ*B signaling and noncanonical NF-*κ*B signaling are required for Th9 cell differentiation and production of inflammatory cytokine IL-9 [11, 15]. On the other hand, NF-*κ*B subunit c-Rel binds to CNS3 and plays a central role in thymic and peripheral Foxp3 expression and Treg cell differentiation [98]. Moreover, NF-*κ*B p65 and c-Rel maintain Treg cells identity and function through directly affecting the expression of Treg lineage-specific transcriptional genes including *Ikzf2* (Helios), *Lrrc32* (GARP), and *Ctla4* [99, 100]. Both *Foxp3* and *Il9* loci can be regulated by costimulatory molecule signals [11, 15]. Under iTreg polarizing conditions, GITR stimulation significantly inhibited the binding of SMAD3 to the *Foxp3* locus and increased the binding of SMAD3 to the *Il9* locus [11]. Using ChIP assays found that GITR stimulation induced NF-*κ*B p50, which recruited histone deacetylase 1 (HDAC1) and sirtuin1 (SIRT1) to the *Foxp3* locus where they mediated histone deacetylation and consequently the inhibition of Foxp3 expression and Treg cell development. Conversely, p50 can activate STAT6, which induces histone hyperacetylation at the *Il9* locus by recruiting p300 histone acetyltransferase, and consequently the induction of Th9 cells. Interestingly, Foxp3 acts as a potent repressor of Th9 cells. Once Foxp3 is induced, it binds to the *Il9* locus and through recruiting histone deacetylases to inhibit transcription of IL-9 and development of Th9 cells [11]. OX40 is another T cell costimulatory molecule in the TNFR superfamily besides GITR [101]. Under Th9 polarizable conditions, OX40 can activate ubiquitin ligase TRAF6, which mediates activation of NF-*κ*B-inducing kinase (NIK). NIK activates the noncanonical NF-*κ*B p52-RelB pathway, which subsequently triggers IL-9 transcription and Th9 cell generation [15].

#### 2.5.5. The Mechanistic Target of the Rapamycin (mTOR) Signaling Pathway

The mechanistic target of rapamycin (mTOR) is a conserved serine/threonine kinase that has central roles in the cell metabolism and growth. mTOR exists in two functionally distinct multiprotein complexes in metazoans, including mTOR complex 1(mTORC1) and mTOR complex 2 (mTORC2) [102]. The mTORC1- and mTORC2-dependent metabolic programming might have cell context-dependent effects on the development and function of Th9 cells and Treg cells [18]. It has been shown that mTORC1 signaling positively regulated Th9 cell differentiation through multiple mechanisms, including increase of hypoxia-inducible factor 1 alpha (HIF1*α*) expression, regulation of histone acetylation at the *Il9* promoter region, and dephosphorylation of Foxo1 [103, 104]. Wang et al. reported that HIF1*α* could directly regulate IL-9 expression through binding to the *Il9* promoter. Modulation of mTORC1-HIF1*α* signaling coupled with glycolytic metabolism promoted Th9 cell differentiation [104]. The mTORC2 signaling also promotes development of Th9 cells and Th9 cell-associated allergic airway inflammation. In an allergic airway inflammation mouse model, mice specifically lacking Rictor (the core component of mTORC2) in CD4^+^T cells showed less Th9 cells and decreased allergic response. Rictor deficiency significantly impaired Th9 cell differentiation through downregulation of IRF4, STAT6 expression, and Akt activity, which indicated that mTORC2 signaling regulated Th9 cell differentiation by modulating IRF4 downstream of either Akt or STAT6 [105]. In contrast, mTORC2-Akt signaling can inhibit Foxp3 expression and consequently suppress the generation and function of iTregs by antagonizing the function of SMAD3 and SMAD4 downstream of TGF-*β*R signaling and by inducing the nuclear exclusion of Foxo1 and Foxo3, respectively [106, 107]. A recent study found that mTORC1 functioned downstream of antigenic signals to promote IRF4, GATA-3 expression, and mitochondrial metabolism and consequently regulated development, homeostasis, and suppressive function of activated Treg cells [108].

#### 2.5.6. The Notch Signaling Pathway

Notch is a receptor that responds to cell surface-bound ligand molecules including Jagged (Jagged1 and Jagged2) and delta-like ligands (DLL1, DLL3, and DLL4) [109]. Notch signaling controls development and differentiation of Th cells [110]. Conditional deletion of Notch1 and Notch2 receptors decreased IL-9 production in Th9 cells differentiated under IL-4 plus TGF-*β*1 conditions [31]. Activation of Notch signaling by Jagged2 ligation promoted Th9 cell differentiation. The Notch1 intracellular domain (NICD1) recruited SMAD3 together with recombining binding protein- (RBP-) J*κ* to bind to the *Il9* promoter and induced its transactivation [31]. Moreover, Notch showed a CD4^+^T cell intrinsic role in promoting IL-4 expression by transactivation of the *Gata*-3 gene [111]. By contrast, activation of Notch signaling by Jagged1 is required for Treg cell lineage commitment, expansion, and maintenance [112]. Notch signaling through its ligand DLL4 promotes iTreg cell differentiation and stability through a MYDN domain-containing protein 3- (SMYD3-) mediated epigenetic mechanism [113]. During allergic airway inflammation, whilst Jagged1-derived signals preferentially induce Th2 response, DLL4 has been shown to enhance Treg cell differentiation and ameliorate the allergic response [114, 115].

Notably, the crosstalk among these signaling cascades might be a more complicated mechanism through which the balance of Th9 and Treg cells is regulated [116]. The balance of these signaling pathways may influence the generation, function, and balanced responses of Th9 and Treg cells [115, 117]. Regulation of the reciprocal developmental signaling pathways in these cells may represent a practical strategy for combating allergic asthma and cancer.

### 2.6. The Instability and Plasticity of Th9 Cells and Treg Cells

Among CD4^+^T cell subsets, Th9 cells and Treg cells seem to exhibit high instability and plasticity under appropriate environmental stimuli [24, 118]. Environmental cues are transmitted to Treg cells and Th9 cells for their functional reprogramming. A study using an *Il9* fate reporter mouse model and in vitro-generated IL-9 expressing T cells demonstrated that IL-9 production was transient and few of the former IL-9-secreting cells maintained IL-9 production [119]. Another study implicated that restimulation of Th9 cells in vivo by exposure to their cognate antigen resulted in diminished IL-9 production [120]. Moreover, depending on the local cytokine milieu present in particular disease models in vivo, adoptively transferred Th9 cells can be reprogrammed into other Th cell subtypes to improve and facilitate adaptive immunity [121]. On the other hand, the instability of Foxp3 in Treg cells has been widely observed and is inherent in their developmental origin [118, 122]. Treg cells manifest functional instability including losing their expression of Foxp3 and suppressive effectiveness, further differentiating into effector Th cells and secreting proinflammatory cytokines in the contexts of infection, organ-specific autoimmunity, and different tumor microenvironments [6, 123–125]. Strikingly, Foxp3^+^Treg cells are also capable of expressing and producing IL-9 in response to inflammatory environmental cues [126]. Murine Th9 cells also produce IL-10 with deficiency of immune-suppressive capability [19, 120, 127]. It has been showed that the costimulatory signal might induce transdifferentiation of Treg cells into Th9 cells. GITR signaling can convert iTregs into Th9 cells by controlling chromatin remodeling at the *Foxp3* and *Il9* loci [128]. The aberrant plasticity and instability of Th9 cells and Treg cells accompanied by changing the effector function might implicate a possible mechanism that affects the balance of Th9 cells and Treg cells in asthma and cancer.

## 3. Roles of Th9 Cells and Treg Cells in Asthma and Cancer

### 3.1. The Role of Proinflammatory Th9 Cells in Asthma

**Figure 4:**
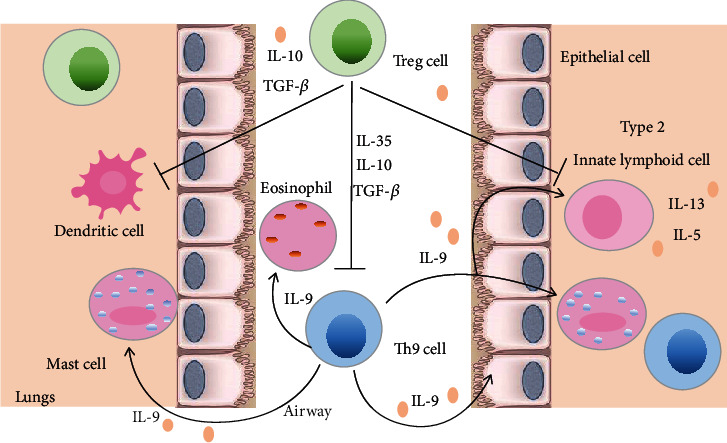
Th9 cells and Treg cells in allergic asthma. Th9 cells and IL-9 could promote activation and accumulation of mast cells (MCs), dendritic cells (DCs), type 2 innate lymphoid cells (ILC2s), and eosinophils, as well as production of IL-13 and IL-5 in asthma. Furthermore, IL-9 and Th9 cells can alter epithelial cell function and promote airway hyperresponsiveness (AHR), mucus hypersecretion, and airway collagen deposition. Inhibition of immune responses mediated by effector T (Teff) cells and innate immune cells is a primary function of Treg cells in asthma. Treg cells restrain Th9 cell, DC, and ILC2 function in the lung, resulting in suppression of inappropriate immune responses. Treg cells also release inhibitory cytokines such as IL-10, TGF-*β*, and IL-35 to control allergic responses.

Many studies have demonstrated that Th9 cells were highly proinflammatory effector T (Teff) cells [5, 129]. Th9 cells are strongly associated with the induction and development of allergic airway inflammation in asthmatic patients and mouse models of asthma [13]. Asthmatic patients have been shown to have increased numbers of Th9 cells in peripheral blood mononuclear cells (PBMCs) and increased IL-9 levels in bronchoalveolar lavage fluid (BALF) [130, 131]. Mouse models of asthma showed increased numbers of IL-9 expressing infiltrating T cells in the lungs when exposed to allergens including house dust mites (HDM), aspergillus, ovalbumin (OVA), and papain [26, 90, 132]. Transfer of OVA-specific Th9 cells into T cell-deficient mice led to severe allergic inflammation after challenge with OVA. Moreover, when these recipient mice were additionally treated with an anti-IL-9 antibody, many of the inflammatory features were blocked [95]. The effector functions of Th9 cells in allergic airway inflammation can be attributed to not only the production of the signature cytokine IL-9 but also the utilization of other lymphoid cell types which may have various roles in allergic asthma [36, 37]. Th9 cells and IL-9 could promote activation and accumulation of MCs, T cells, type 2 innate lymphoid cells (ILC2s), and eosinophils concomitantly with increased levels of IgE, IL-13, and IL-5 in asthma models [36, 37] (Figure 4). Importantly, various cytokines, TFs, TCR, and costimulatory signals are involved in Th9 cell-mediated allergic immunopathology. Studies using adoptively transferred models of allergic asthma demonstrated that exogenously or ectopically expressed proallergic cytokines such as TSLP, TNF family cytokine TLI1 (Tnfsf15), and IL-25 promoted airway inflammation by increasing Th9 cell differentiation and IL-9 production [29, 133, 134]. The Tec family tyrosine kinase Itk is a component of TCR-mediated signaling. In an allergic asthma model, mice with a deficiency of Itk showed reduced pulmonary inflammation and IL-9 production by T cells. The in vitro experiment further demonstrated that Itk induced Th9 differentiation by regulating IRF4 [132]. Similarly, allergic inflammation was attenuated in mice with deficiencies in specific TFs including PU.1 and BATF [48, 135]. OX40 costimulatory signaling has been demonstrated to promote allergic airway inflammation by inducing Th9 cell generation [15]. Furthermore, the airway microenvironment in asthma might further amplify Th9 cell-mediated immune response and allergic inflammation. During allergic asthma, inflammatory cytokines induce nitric oxide synthase (NOS) activity in the airway epithelium and inflammatory cells resulting in the production of large amounts of nitric oxide (NO) [136]. NO can enhance Th9 cell differentiation by increasing p53-mediated IL-2 production, STAT5 phosphorylation, and IRF4 expression resulting in developing more severe airway inflammation [137]. These data indicate important roles of cytokines, TFs, TCR signaling, costimulatory molecules, and airway microenvironment in regulating development and differentiation of Th9 cells to promote development of asthma. Zhao et al. recently showed that A 7 amino acid peptide (7P) of the hypervariable region 1 (HVR1) of hepatitis C virus could inhibit Th9 differentiation and IL-9 production by blocking CD81 signaling in an OVA-induced asthma model [138].

### 3.2. The Role of Anti-inflammatory Treg Cells in Asthma

The pathogenesis of allergic asthma entails a dysfunctional tolerogenic immune response towards allergens [14]. Genetic and immunological evidence indicates that Treg cells play pivotal roles in promoting tolerance to allergens and preventing allergic asthma [139]. Moreover, a number of studies have investigated associations of impaired function and decreased frequencies of Treg cells with the development of allergic asthma [140–142]. Adoptive transfer of antigen-specific Treg cells suppressed allergic inflammation and airway hyperreactivity in asthmatic mice [143]. The precise mechanisms by which Treg cells employ to suppress allergic immune responses in asthmatic chronic inflammatory environments are complex. Prevention of Teff cells and innate immune cell-mediated immune responses is one of the primary functions of Treg cells in asthma [144]. Treg cells can restrain DC and ILC2 function in the lung, resulting in suppression of inappropriate immune responses [145]. Moreover, another inhibitory mechanism utilized by Treg cells appears to be via anti-inflammatory cytokines such as IL-10, TGF-*β*1, and IL-35 [146–148] (Figure 4). Interestingly, evidence suggests that the capacity of Treg cells to suppress the effect Th cells responses can be associated with the acquisition or alteration of specific TFs and cytokines [6, 149]. In the course of regulating Th cell immune responses, Treg cells can partially mimic the phenotype of the Teff cells by expressing their master TFs. This capacity could endow Treg cells with finely tuned homing, survival, and functional properties [150]. For instance, IRF4 expression endows Treg cells with the ability to suppress Th2 responses [6]. Importantly, studies of immune dysregulation, polyendocrinopathy, and enteropathy X-linked (IPEX) which are characterized by rare human autoimmune disorder and allergic inflammation elucidated that Foxp3 was specifically required for Treg cell function in asthma [151]. Foxp3-deficient mice exhibit a proallergic phenotype associated with Th2 cell-induced airway inflammation and elevated serum levels of IgE and IgA [152].

### 3.3. The Antitumor Effect of Th9 Cells

**Figure 5:**
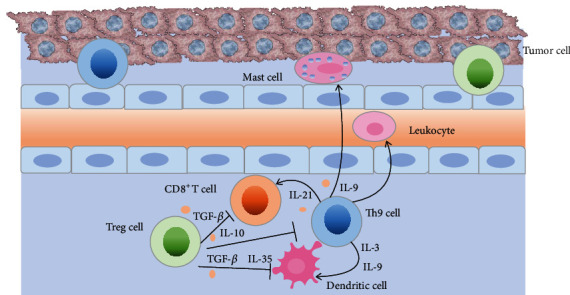
Th9 cells and Treg cells in cancer. The mechanisms underlying the antitumor effects of Th9 cells seem to be mainly dependent on the secretion of IL-9, IL-21, and IL-3 and activation of different types of immune cells such as MCs, DCs, and CD8^+^T cells. IL-9 can induce recruitment of DCs and leukocytes. Then, DCs activate CD8^+^T cells through the crosspresentation of tumor antigens, which facilitates tumor rejection. IL-3 and IL-21 have also been shown to promote survival of DCs and enhance response of CD8^+^T cells. Production of TGF-*β*, IL-10, and IL-35 by Treg cells prevents full cytotoxic effector differentiation in tumor-specific CD8^+^T cells and suppresses Teff cells such as Th9 cell differentiation and function. Treg cells that expand during tumor progression contribute to the immune tolerance of cancer by impeding the ability of DCs to orchestrate immune response.

In recent years, several groups have reported that Th9 cells exhibit potent antitumor activity [153]. The adoptive transfer of antigen-specific Th9 cells could prevent tumor progression in diverse animal models for human cancer [39, 71]. The mechanisms underlying the antitumor function of Th9 cells are most probably dependent on their secretion of IL-9, IL-21, IL-3, and granzyme B (GzmB) and activation of different types of immune cells such as MCs, DCs, and CD8^+^T cells [50, 71, 154, 155]. IL-9 could induce recruitment of DCs and leukocytes via the CC-chemokine ligand 20- (CCL20-) CC-chemokine receptor 6 (CCR6) axis and promote DCs to migrate to tumor-draining lymph nodes (TDLNs). Then, DCs activated CD8^+^T cells in the TDLNs through the crosspresentation of tumor antigens, which facilitated tumor rejection [156]. IL-3 and IL-21 have also been shown to promote survival of DCs and enhance response of CD8^+^T cells, respectively [71, 157] (Figure 5). Moreover, specific cytokines, TFs, TCR signaling, and costimulatory signals are involved in antitumor function of Th9 cells. GITR costimulation mediated antitumor immunity by promoting Th9 cell differentiation and tumor-specific CTL responses as well as DC activation in a model of colon carcinogenesis [38]. Autophagy induction following TCR signaling activation was shown to selectively restrain Th9 cell polarization and to induce ubiquitination and degradation of PU.1, resulting in the repression of antitumor activity of Th9 cells [158]. Under Th9 polarizing conditions, IL-1*β* enhanced IL-9 and IL-21 production from Th9 cells by inducing phosphorylation of STAT1 and subsequent expression of IRF1, which are bound to the promoters of *Il9* and *Il21* [71]. IL-1*β* combined with IL-4 induces a Th9^IL-4+IL-1*β*^ cell subset. When compared with classic Th9^IL-4+TGF-*β*^ cells, Th9^IL-4+IL-1*β*^ cells were less exhausted and exhibited a cytotoxic T effector gene signature and a superior antitumor effect in a mouse melanoma model [159]. Importantly, the tumor microenvironment (TME) might affect Th9 cell differentiation and responses. TME is adenosine triphosphate (ATP) rich [160]. Roy and Awasthi showed that the extracellular ATP (eATP) induced the production of NO, which potentiated the mTOR-HIF1*α*-mediated metabolic signaling pathway to promote differentiation of human Th9 cells and secretion of IL-9 [161].

### 3.4. The Protumor Effects of Treg Cells

Treg cells are aberrantly enriched in tumors, where they can dampen antitumor immune responses and promote tumor development and progression [162]. The protumor effects of Treg cells are proved by any amount of preclinical data in which systemic or selective depletion of Treg cells or inhibition of their function increases antitumor-specific immune responses and reduces tumor burden [94, 163–165]. A study using transcriptome analysis and single-cell sequencing showed that tumor-infiltrating Treg cells were highly suppressive and expressed with high-frequency genes that were associated with increased suppressor activity, such as ICOS, OX40, GITR, CTLA-4, and PD-1 [165]. Furthermore, immune checkpoint receptors (ICR) including PD-1, PDL1, and immunosuppressive molecules such as CD39 and LAP are also implicated in the induction and function of Treg cells in cancer [166, 167]. Tumor-infiltrating Treg cells in mice and humans predominantly express C-C chemokine receptor 4 (CCR4), which contributes to recruitment of Treg cells to tumors [168]. Production of TGF-*β*, IL-10, and IL-35 by Treg cells prevents full cytotoxic effector differentiation in tumor-specific CD8^+^T cells and suppresses other immune cells differentiation and function [169, 170]. Treg cells that expand during tumor progression contribute to the immune tolerance of cancer by impeding the ability of DCs to orchestrate immune responses [171] (Figure 5). Treg cells suppress Teff cell function within tumors [171]. Treg/Teff ratios have been suggested to correlate with effective antitumor responses [172]. Low Treg-cell-to-Teff-cell ratios are associated with favorable survival in various types of human tumors [162]. In murine tumor models, transient depletion of Treg cells led to activation of CD4^+^ or CD8^+^T cells and rejection of solid tumors [173, 174]. Liu et al. recently showed that human Treg cells initiated DNA damage response and cellular senescence in Teff cells caused by metabolic competition during crosstalk, resulting in Teff cell functional changes in NOD-scid IL2R*γ*^null^ mice. Furthermore, TFs STAT1/STAT3 and extracellular signal-regulated kinase (ERK1/2) and p38 mitogen-activated protein kinase (p38 MAPK) signaling were also involved in Teff cell senescence induced by Treg cells [175]. Importantly, specific TFs, cytokine milieu, TCR, and costimulatory signals might regulate function and differentiation of Treg cells in tumor development. Tumor-infiltrating Treg cells with increased expression of IRF4 were highly activated and preferentially expanded in lung, liver, and melanoma tumors compared with the adjacent normal tissues. Deletion of IRF4 in Treg cells resulted in enhanced antitumor immunity and delayed tumor growth in a mouse model of cancer [94].

## 4. Molecular Mechanisms That Influence the Balance between Th9 Cells and Treg Cells in Asthma and Cancer

Th9 cells and Treg cells have been demonstrated to function simultaneously in the setting of allergic asthma and cancer [11, 12, 15]. They share key TFs and common induction pathways. An interaction of complex inflammatory and tumor microenvironmental cues with other factors including TFs, cytokine signals, costimulatory molecules, and metabolic pathways regulates differentiation and function of Th9 cells and Treg cells [18, 176–178]. Although promising results have been achieved with Treg cell depletion strategies, severe autoimmunity, allergic inflammation, and chronic infection may occur following systemic depletion of Treg cells or Th9 cells [179–181]. Thus, a more detailed understating of molecular mechanisms underlying the balance between Treg cells and Th9 cells will help establish new strategies for the treatment and prevention of asthma and cancer.

### 4.1. TFs and Cytokine Signals Involved in the Balance between Th9 Cells and Treg Cells in Asthma and Cancer

**Figure 6:**
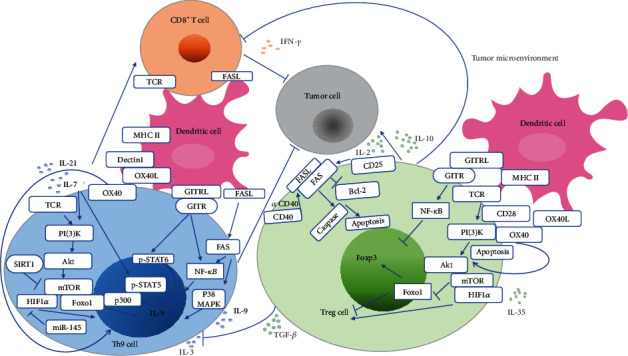
Signaling pathways critical for the roles and differentiation of Th9 cells and Treg cells in solid tumor. Treg cells and Th9 cells receive signals through surface receptors such as TCR, CD28, and costimulatory molecules GITR and OX40, interacting with ligands on neighboring cells such as DCs and CD8^+^T cells. Engagement of the TCR complex and of the coreceptor CD28 activates the phosphoinositide 3-kinase- (PI (3) K-) Akt and mTOR pathways, which inactivate Foxo1. Expression of the Akt-insensitive Foxo1 mutant depletes tumor-associated activated Treg (aTreg) cells and activate CD8^+^T cell response in the mammary tumor model. In the mouse melanoma model, PI (3) K-Akt-mTOR signaling is activated by IL-7, which results in nuclear accumulation of Foxo1 and activation of STAT5 that binds to the p300 promoter to induce differentiation of Th9 cells and production of IL-9. OX40/OX40L engagement activates the AKT-mTOR pathway, which induces phosphorylation and nuclear exclusion of Foxo1 to inhibit Foxp3 induction. Activation of OX40 induces Treg cell apoptosis and increases the CD8^+^T cell/Treg cell ratio. OX40L can be induced by the dectin-1 signaling pathway. Dectin-1 activates DCs and induces a potent antitumor response that is dependent on Th9 cells and CD8^+^T cells. Activation of GITR by its ligand upregulates NF-*κ*B to induce a repressive chromatin structure of the *Foxp3* locus and consequently inhibit Treg cell differentiation. GITR/GITRL engagement improves antitumor activity and differentiation of Th9 cells by activating the NF-*κ*B pathway and STAT6/p300. GITR/GITRL engagement also promotes IL-9-dependent enhancement of CD8^+^T cytotoxic T lymphocyte (CTL) response and increases the crosspresentation capacity of DCs. FAS/FAS ligand (FASL) signaling activates NF-*κ*B and p38 mitogen-activated protein kinase (p38 MAPK) to promote Th9 cell differentiation, as well as IFN-*γ* producing CD8^+^T cell generation to enhance antitumor immunity in the Lewis lung carcinoma model. Activation of Fas by IL-2 and agonistic CD40 antibody (*α*CD40) results in Treg cell apoptosis in the renal adenocarcinoma (Renca) tumor model. Inhibition of the mTOR-hypoxia-inducible factor 1*α* (HIF1*α*) signaling pathway by histone deacetylase sirtuin1 (SIRT1) negatively regulates differentiation of Th9 cells in the melanoma tumor model. Inhibition of HIF1*α* by microRNA miR-145 suppresses differentiation of Th9 cells in the liver cancer malignant ascites model. HIF1*α* endows Treg cells with metabolic property that allows them to endure within the tumor. Specific ablation of HIF1*α* in Treg cells results in enhanced CD8^+^T cell suppression in the glioblastoma tumor model.

The Foxo family of TFs is required for the regulation of T cell activation and differentiation [182, 183]. Foxo1 and Foxo3 are regulated by the phosphoinositide 3-kinase- (PI (3) K-) Akt pathway, in which Akt kinases are activated by PI (3) K phosphorylation of Foxo1 and Foxo3, leading to their inactivation and nuclear exclusion [184]. Moreover, P1(3) K/Akt and mTOR signaling pathways can be activated by TCR and CD28 and lead to inactivate Foxo1 [185]. Foxo1 and Foxo3 are highly expressed in Treg cells [186]. The study using mice with T cell-specific deletion of Foxo1 and Foxo3 alleles found that *Foxo1^−/−^Foxo3^−/−^* mice developed splenomegaly and lymphadenopathy at 8 weeks of age. Heavy mononuclear cell infiltration of various vital organs, including the lung, liver, and colon, was also observed in *Foxo1^−/−^Foxo3^−/−^* mice. This finding demonstrated the critical roles of T cell Foxo1 and Foxo3 in the protection of mice from inflammatory disorders. Mechanistically, Foxo1 and Foxo3 binding to the *Foxp3* CNS1 region led to regulate Foxp3 transcription, as well as Treg cell differentiation and function [187]. By contrast, a recent study found that differentiation of activated Treg (aTreg) cells was associated with repression of Foxo1-dependent gene transcription, concomitant with reduced Foxo1 expression and enhanced Foxo1 phosphorylation at sites of the Akt kinase. aTreg cells were mainly present in nonlymphoid tissues including solid tumors and had a crucial function in suppressing CD8^+^T cell responses. In the spontaneous MMTV-PyMT mammary tumor model, expression of the Akt-insensitive Foxo1 mutant at a low dose was sufficient to deplete tumor-associated aTreg cells and promote CD8^+^T cell responses, resulting in inhibition of tumor growth [163]. Moreover, Bi et al. reported that B16F10-OVA tumor-bearing mice treated with IL-7-pretreated Th9 cells had a decreased tumor burden. IL-7 increased activation of Foxo1 and the abundance of the histone acetyltransferase p300 by activating PI (3) K-Akt-mTOR signaling and STAT5. p300 acted as a coactivator to enable Foxo1 to bind to the *Il9* promoter to induce IL-9 transcription, resulting in the differentiation of Th9 cells and production of IL-9 protein [103] (Figure 6). Interestingly, the in vitro experiment demonstrated that Foxo1 was critically required for IL-9 induction in both Th9 cells and iTreg cells by binding to *Il9* and *Irf4* promoters, which were crucial for IL-9 induction in these cells. Inhibition of PI (3) K-AKT enhanced IL-9 production in Th9 cells via Foxo1. Furthermore, therapeutic silencing of Foxo1 by siRNA in OVA-induced asthmatic mice reduced accumulation of infiltrating inflammatory cells around the bronchi and vessel, as well as decreased bronchial hyperplasia, and IL-9 production in BALF, when compared to OVA-challenged Scr-siRNA-treated mice [54]. These findings suggest that the Foxo1 signaling pathway may regulate the balance of Treg cells and Th9 cells. Regulation of the Foxo1 signaling pathway may be beneficial in the treatment of asthma and cancer.

The STAT5 activity is a common gateway to the programming of differentiation of Th9 cells and Treg cells [78, 188]. IL-2, a growth factor capable of driving the development of activated T cells, functions through STAT5 [69]. Recent studies found that activated STAT5 could directly bind to the promoter of the *Il9* locus and induce IL-9 transcription [53, 92]. In different asthma models, the IL-2-STAT5 pathway is regulated by various factors including NO, TL1A, and Itk and contributes to induction of IL-9 and differentiation of Th9 cells [29, 132, 137]. Furthermore, STAT5 also stabilizes Foxp3 expression and induces differentiation of Treg cells by binding to the *Foxp3* CNS2 region [76]. Treg cells and Th9 cells are commonly regulated by the TSLP-STAT5 pathway in the context of allergic inflammation. TSLP activates STAT5 to promote IL-9 production and differentiation of Th9 cells in vitro. In vivo using an adoptive transfer model demonstrated that TSLP was able to promote allergic airway inflammation by enhancing the Th9 cell response [134]. In another study, Treg cells were isolated from BALF samples in allergic asthmatic, healthy control, and nonallergic asthmatic subjects to evaluate the influence of TSLP on immunosuppressive activities of Treg cells and its potential consequences in human allergic asthma. Activated pulmonary Treg cells expressing TSLP-R responded to TSLP-mediated activation of STAT5 and exhibited a significant decrease in suppressive activity and IL-10 production compared to healthy control and their nonallergic asthmatic counterparts [189]. Thus, regulation of Th9 cell and Treg cell balance by IL-2-STAT5 and TSLP-STAT5 signaling pathways may present insights into novel therapeutic strategies to control asthma.

On the other hand, the IL-2-Janus kinase 3- (JAK3-) STAT5 pathway is negatively regulated by transcription factor B cell lymphoma 6 (Bcl6), which competes with STAT5 for binding to the *Il9* promoter of Th9 cells and directly represses the *Il9* gene [92]. Ogasawara et al. showed that the inhibitory function of Bcl6 in naturally occurring memory phenotype CD4^+^T cells attenuated allergic airway inflammation [190]. Moreover, the transfer of Bcl6-deficient (Bcl6^−/−^) Treg cells into asthmatic mice failed to suppress Th2 responses in allergic airway inflammation [191, 192]. Bcl6^−/−^ Treg cells also displayed increased levels of GATA-3, which suggested that Bcl6 was required to suppress Th2 genes in Treg cells by repressing GATA-3 transcriptional transactivation [192]. A recent study found that Bcl6 was essential in maintaining the lineage stability of Treg cells in TME and deletion of Bcl6 in Treg cells resulted in impaired suppressive function and tumor regression [193]. Bcl6 has therefore been proposed to be a regulator that modulates the balance between Treg cells and Th9 cells in asthma and cancer.

### 4.2. Costimulatory Molecules Involved in the Balance between Th9 Cells and Treg Cells in Asthma and Cancer

OX40 is expressed on both activated Treg cells and Th9 cells. OX40/OX40 ligand (OX40L) engagement delivers a potent costimulatory signal to activated Th9 cells and Treg cells and regulates their survival, differentiation, and function [15, 194]. Xiao et al. found that OX40 signaling induced super-enhancer (SEs) formation at the *Il9* locus through RelB/p300-mediated chromatin acetylation to promote the induction of Th9 cells and IL-9 expression. The agonist antibody-targeting OX40 (OX86) and BET protein inhibitor JQ1 which acts primarily by disrupting SEs exhibit potency in promoting and suppressing allergic airway inflammation, respectively, in OVA-induced mice [195]. Zhang et al. found that OX40 upregulated BATF3 and BATF, which produced a closed chromatin configuration at the *Foxp3* locus to repress Foxp3 expression by recruiting the histone deacetylase Sirt1/7 in activated CD4^+^T cells. Moreover, OX40 inhibits Foxp3 expression and iTreg cell induction by activating the AKT-mTOR pathway, which induces phosphorylation and nuclear exclusion of Foxo1 [196]. The combined use of cyclophosphamide (CTX) and an agonist antibody targeting the OX86 could enhance antitumor immunity and was capable of regressing established B16 melanoma tumors by inducing tumor-infiltrating Treg cell apoptosis and favorably increasing the intratumoral CD8^+^T cell/Treg cell ratio [197]. Zhao et al. found that dectin-1 signaling stimulated DCs to overexpress surface costimulatory molecule ligand OX40L, which was required to promote Th9 cell differentiation. Immunization of B16F10-OVA tumor-bearing mice with dectin-1-activated DCs induced potent antitumor response that was dependent on induction of Th9 cells and IL-9 production [155]. These findings provide insights on OX40 in the control of Treg cells and Th9 cell balance and may provide treatment strategies for asthma and cancer.

GITR costimulatory signaling is involved in the regulation of the balance of Treg cells and Th9 cells in the TME. Studies using an agonist anti-GITR antibody have shown a negative role for GITR signaling in Foxp3^+^ Treg cell differentiation [11]. Moreover, activation of GITR signaling induced Foxp3^+^ Treg cells to transdifferentiate into Th9 cells. GITR-induced IL-9 promoted tumor-specific CD8^+^ cytotoxic T lymphocyte (CTL) response by upregulating the expression of costimulatory and MHC II molecules and increasing the crosspresentation capacity of GITR ligand- (GITRL-) expressing DCs in a melanoma tumor model. Mechanistically, GITR signaling enhanced the TRAF6-NF-*κ*B pathway and activated STAT6, which rendered the *Il9* locus accessible through recruitment of histone acetyltransferase p300. GITR upregulated NF-*κ*B family member p50 to recruit and position histone deacetylases to the *Foxp3* locus to induce a repressive chromatin structure [11, 12, 38]. Thus, therapeutic approaches targeting the GITR signaling pathway may provide additional means of therapeutic intervention in cancer.

Fas (CD95), another member of the TNFR family, plays important roles in T cell differentiation and activation [198, 199]. In Lewis lung carcinoma OVA-bearing mice, Fas costimulatory signaling could promote Th9 cell differentiation and IL-9 production, as well as IFN-*γ* producing CD8^+^T cell generation to enhance antitumor immunity. Mechanistically, Fas signaling activated PKC*β*, which induced activation of NF-*κ*B and p38 MAPK to promote Th9 cell differentiation [200]. Moreover, IL-2 and agonistic CD40 antibody (*α*CD40) treatment induced Fas-mediated elimination of Treg cells in the renal adenocarcinoma (Renca) tumor-bearing mice. Fas ligand (FasL) expressed by CD8^+^T cells binding to Fas expressed on Treg cells can activate caspases and downregulate Bcl-2 expression, resulting in Treg cell apoptosis in the TME [201]. Fas may affect the balance between Th9 cells and Treg cells in cancer.

### 4.3. Metabolic Signaling Involved in the Balance between Th9 Cells and Treg Cells in Asthma and Cancer

Distinct metabolic programs support the differentiation of Th cells into their separate functional subsets [202]. It has been demonstrated that Th9 cells are strongly dependent on glycolysis, whereas Treg cells depend more on the oxidation of lipids [104, 203]. NAD^+^- (nicotinamide adenine dinucleotide-) dependent lysine deacetylase SIRT1 has emerged as a key metabolic sensor that couples energy metabolism to transcriptional regulation of inflammation [204]. SIRT1 can inhibit differentiation of Th9 cells by decreasing glycolytic activity, and its deficiency promotes Th9 cell differentiation through a mTOR1-HIF1*α*-dependent glycolytic pathway. A recent study establishing an OVA-induced allergic airway inflammation model in wild-type (WT) and CD4^+^T cell-specific SIRT1-deficient (*Sirt1*^flox/flox^*Cd4-Cre*) mice demonstrated that *Sirt1*^flox/flox^*Cd4-Cre* mice exhibited more severely pathogenic lung inflammation and higher IL-9 secretion, compared with WT mice. Moreover, Rag1^−/−^ mice bearing B16 melanoma that received SIRT1-deficient CD4^+^T (*Sirt1*^flox/flox^*Cd4-Cre)* cells exhibited smaller tumors compared with the WT mice that received CD4^+^T cells. Tumor-infiltrating CD4^+^T cells isolated from the *Sirt1*^flox/flox^*Cd4-Cre* mice displayed higher IL-9 production and IL9^+^ ratio accompanied by higher glycolytic activity, compared with CD4^+^T cells isolated from WT [104]. As such, SIRT1-mTOR-HIF1*α* signaling coupled with the glycolytic pathway is required for differentiation of Th9 cells and modulation of Th9 cell-associated allergic airway inflammation as well as tumor regression. Moreover, Kwon et al. showed that SIRT1 was a negative regulator of Treg cell function and Foxp3 expression through deacetylation of lysine residues in *Foxp3* [205]. By contrast, Marcel et al. showed that SIRT1 inhibited the Notch1 intracellular domain to promote the survival and function of activated Treg cells. In mouse models of inflammation, deletion of SIRT1 or Nothc1 decreased Treg cell survival and exacerbated inflammatory diseases [206]. These results provide key mechanistic insights into how SIRT1 regulates the development and function of Th9 cells and Treg cells during allergic airway inflammation and cancerous tumors. SIRT1 signaling may be targeted therapeutically to regulate the Th9 cell and Treg cell balance in asthma and cancer.

The canonical hypoxia sensor HIF1*α* has been demonstrated to regulate differentiation and function of Th9 cells and Treg cells in tumors [207]. A study using glioblastoma-bearing mice that received HIF1*α*-deficient Treg cells has revealed that these mice showed significantly enhanced animal survival and a reduction in Treg cell numbers within the tumors. In the context of hypoxia, HIF1*α* directs glucose away from mitochondria, leaving Treg cells dependent on fatty acids for mitochondrial metabolism within the hypoxic tumor [208]. A study using a liver cancer malignant ascites (MA) model showed that HIF1*α* expression and PI3K/Akt/mTOR/p70 ribosomal protein S6 kinase (p70S6K) pathway activation could be inhibited by microRNA (miR-145), which led to suppression of Th9 differentiation and IL-9 production in MA [209]. HIF1*α* signaling may act as a metabolic regulator for the balance of Treg cells and Th9 cells in tumor (Figure 6).

## 5. Concluding Remarks

Although Th9 cells and Treg cells fundamentally demonstrate opposing function in asthma and cancer, they exert both beneficial and pathogenic impacts depending on a particular biological setting. In addition to favoring of anticancer immunity and tumor elimination, Th9 cells promote tumor development, especially in hepatocellular carcinoma and lung cancer [178, 210]. The role of Treg cells in cancer is also ambiguous, as they are critical inhibitory regulators in solid tumors; whereas during inflammation-induced tumorigenesis, they prevent cancer initiation by restraining inflammation [211, 212]. Moreover, during the severity of allergic airway inflammation and tumor, Treg cells adapt to the local environmental changes through functional and phenotypic reprogramming [213, 214]. High-grade gliomas have more regions under chronic hypoxia than lower-grade tumors [215]. Treg cells may become unstable and plastic under hypoxia [216]. Intense inflammation in experimental allergic asthma may lessen Treg cell suppression or redirect Treg cells to proinflammatory phenotypes [213, 217, 218]. Although Th9 cells have great capacity to respond to changing environments such as cytokine environments and inflammatory environments and acquire modified gene expression patterns and function [19, 39, 120], there is little study of functional and phenotypic reprogramming of Th9 cells in severity of asthma and tumor. Meanwhile, most research focused on exploring mechanisms by which various factors including cytokine signals, transcriptional factors, and metabolic cues regulate development, differentiation, and function of Th9 cells and Treg cells in response to inflammatory or tumor conditions. What has received less attention, however, is the balance between these cells in asthma and cancer. Considering the complex interplay between Treg cells and Th9 cells on allergic and tumor immunity, it remains significant to recognize the mechanisms for the regulation of the function, stability, and balance of these cells in response to a constantly changing inflammatory and tumor microenvironment, which may influence the development and outcome of asthma and cancer. Importantly, further elucidation of the cellular and molecular processes underlying complexity interplay of Th9 cells and Treg cells will help in providing therapeutic strategies to selectively suppress or enhance immune responses in asthma and cancer by controlling the balance between Th9 cells and Treg cells.

## Figures and Tables

**Table 1:** Specific cytokines, signal transducer, activator of transcription (STAT) members, and transcription factors (TFs) in the development and differentiation of Th9 cell and Treg cell.

Subset	Cytokines	STAT members	Master TFs	Other TFs
Th9	IL-2, IL-4, TGF-*β*, IL-1, IL-25	STAT6, STAT5	?	IRF4, PU.1, BATF, GATA-3, NFAT, SMAD 2/3/4, NF-*κ*B RelB/p52, Foxo1, ETV5, IRF1
Treg	TGF-*β*, IL-2	STAT5	Foxp3	Foxp1, AML1/Runx1, CREB, Foxo1, NFAT, c-Maf, Blimp-1, BATF, IRF4, Eos, Myb, GATA-3, JunB, SMAD 2/3, NF-*κ*B p65/c-Rel
Th9, Treg	TGF-*β*, IL-2	STAT5		IRF4, BATF, SMAD 2/3, NFAT, GATA-3, Foxo1

## Data Availability

The data used to support the findings of this study are included within the article.

## References

[1] Littman D. R., Rudensky A. Y. (2010). Th17 and regulatory T cells in mediating and restraining inflammation. *Cell*.

[2] Elkord E., Sasidharan Nair V. (2018). T-regulatory cells in health and disease. *Journal of Immunology Research*.

[3] DuPage M., Bluestone J. A. (2016). Harnessing the plasticity of CD4^+^ T cells to treat immune-mediated disease. *Nature Reviews Immunology*.

[4] Boyton R. J., Altmann D. M. (2002). Is selection for TCR affinity a factor in cytokine polarization?. *Trends in Immunology*.

[5] Kaplan M. H., Hufford M. M., Olson M. R. (2015). The development and *in vivo* function of T helper 9 cells. *Nature Reviews Immunology*.

[6] Zheng Y., Chaudhry A., Kas A. (2009). Regulatory T-cell suppressor program co-opts transcription factor IRF4 to control T_H_2 responses. *Nature*.

[7] Ohkura N., Kitagawa Y., Sakaguchi S. (2013). Development and maintenance of regulatory T cells. *Immunity*.

[8] Lloyd C. M., Hawrylowicz C. M. (2009). Regulatory T cells in asthma. *Immunity*.

[9] Sun L., Fu J., Lin S. H. (2020). Particulate matter of 2.5 *μ*m or less in diameter disturbs the balance of T_H_17/regulatory T cells by targeting glutamate oxaloacetate transaminase 1 and hypoxia-inducible factor 1*α* in an asthma model. *The Journal of Allergy and Clinical Immunology*.

[10] Nishikawa H., Sakaguchi S. (2014). Regulatory T cells in cancer immunotherapy. *Current Opinion in Immunology*.

[11] Xiao X., Shi X., Fan Y. (2015). GITR subverts Foxp3^+^ Tregs to boost Th9 immunity through regulation of histone acetylation. *Nature Communications*.

[12] Kim I. K., Chung Y., Kang C. Y. (2016). GITR drives TH9-mediated antitumor immunity. *Oncoimmunology*.

[13] Koch S., Sopel N., Finotto S. (2017). Th9 and other IL-9-producing cells in allergic asthma. *Seminars in Immunopathology*.

[14] Noval Rivas M., Chatila T. A. (2016). Regulatory T cells in allergic diseases. *Journal of Allergy and Clinical Immunology*.

[15] Xiao X., Balasubramanian S., Liu W. T. (2012). OX40 signaling favors the induction of T_H_9 cells and airway inflammation. *Nature Immunology*.

[16] Tamiya T., Ichiyama K., Kotani H. (2013). Smad2/3 and IRF4 play a cooperative role in IL-9-producing T cell induction. *The Journal of Immunology*.

[17] van der Veeken J., Arvey A., Rudensky A. (2014). Transcriptional control of regulatory T-cell differentiation. *Cold Spring Harbor Symposia on Quantitative Biology*.

[18] Wang P., Zhang Q., Tan L., Xu Y., Xie X., Zhao Y. (2020). The regulatory effects of mTOR complexes in the differentiation and function of CD4+ T cell subsets. *Journal of Immunology Research*.

[19] Dardalhon V., Awasthi A., Kwon H. (2008). IL-4 inhibits TGF-*β*-induced Foxp3^+^ T cells and, together with TGF-*β*, generates IL-9^+^ IL-10^+^ Foxp3^−^ effector T cells. *Nature Immunology*.

[20] Wang A., Pan D., Lee Y. H., Martinez G. J., Feng X. H., Dong C. (2013). Cutting edge: Smad2 and Smad4 regulate TGF-*β*–mediatedIl9gene expression via EZH2 displacement. *The Journal of Immunology*.

[21] Angkasekwinai P., Chang S. H., Thapa M., Watarai H., Dong C. (2010). Regulation of IL-9 expression by IL-25 signaling. *Nature Immunology*.

[22] Schmitt E., Beuscher H. U., Huels C. (1991). Il-1 serves as a secondary signal for Il-9 expression. *Journal of Immunology*.

[23] Schmitt E., Germann T., Goedert S. (1994). Il-9 production of naive Cd4(+) T-cells depends on Il-2, is synergistically enhanced by a combination of Tgf-beta and Il-4, and is inhibited by Ifn-gamma. *Journal of Immunology*.

[24] Veldhoen M., Uyttenhove C., van Snick J. (2008). Transforming growth factor-*β* ‘reprograms’ the differentiation of T helper 2 cells and promotes an interleukin 9-producing subset. *Nature Immunology*.

[25] Licona-Limón P., Henao-Mejia J., Temann A. U. (2013). Th9 cells drive host immunity against gastrointestinal worm infection. *Immunity*.

[26] Kerzerho J., Maazi H., Speak A. O. (2013). Programmed cell death ligand 2 regulates T_H_9 differentiation and induction of chronic airway hyperreactivity. *Journal of Allergy and Clinical Immunology*.

[27] Palmer M. T., Lee Y. K., Maynard C. L. (2011). Lineage-specific Effects of 1,25-dihydroxyvitamin D_3_ on the development of effector CD4 T cells. *Journal of Biological Chemistry*.

[28] Mikami N., Miyagi Y., Sueda K. (2013). Calcitonin gene-related peptide and cyclic adenosine 5'-monophosphate/protein kinase A pathway promote IL-9 production in Th9 differentiation process. *The Journal of Immunology*.

[29] Richard A. C., Tan C., Hawley E. T. (2015). The TNF-family ligand TL1A and its receptor DR3 promote T cell-mediated allergic immunopathology by enhancing differentiation and pathogenicity of IL-9-producing T cells. *The Journal of Immunology*.

[30] Jäger A., Dardalhon V., Sobel R. A., Bettelli E., Kuchroo V. K. (2009). Th1, Th17, and Th9 effector cells induce experimental autoimmune encephalomyelitis with different pathological phenotypes. *The Journal of Immunology*.

[31] Elyaman W., Bassil R., Bradshaw E. M. (2012). Notch receptors and Smad3 signaling cooperate in the induction of interleukin-9-producing T cells. *Immunity*.

[32] Malik S., Dardalhon V., Awasthi A. (2017). Characterization of Th9 cells in the development of EAE and IBD. *Methods in Molecular Biology*.

[33] Harusato A., Abo H., Ngo V. L. (2017). IL-36*γ* signaling controls the induced regulatory T cell-Th9 cell balance via NF*κ*B activation and STAT transcription factors. *Mucosal Immunology*.

[34] Schmitt E., Klein M., Bopp T. (2014). Th9 cells, new players in adaptive immunity. *Trends in Immunology*.

[35] Lu Y., Wang Q., Xue G. (2018). Th9 cells eepresent a unique subset of CD4^+^ T cells endowed with the ability to eradicate advanced tumors. *Cancer Cell*.

[36] Moretti S., Renga G., Oikonomou V. (2017). A mast cell-ILC2-Th9 pathway promotes lung inflammation in cystic fibrosis. *Nature Communications*.

[37] Sehra S., Yao W., Nguyen E. T. (2015). TH9 cells are required for tissue mast cell accumulation during allergic inflammation. *Journal of Allergy and Clinical Immunology*.

[38] Kim I. K., Kim B. S., Koh C. H. (2015). Glucocorticoid-induced tumor necrosis factor receptor-related protein co- stimulation facilitates tumor regression by inducing IL-9-producing helper T cells. *Nature Medicine*.

[39] Purwar R., Schlapbach C., Xiao S. (2012). Robust tumor immunity to melanoma mediated by interleukin-9-producing T cells. *Nature Medicine*.

[40] Vegran F., Apetoh L., Ghiringhelli F. (2015). Th9 cells: a novel CD4 T-cell subset in the immune war against cancer. *Cancer Research*.

[41] Chen T., Guo J., Cai Z. (2020). Th9 cell differentiation and its dual effects in tumor development. *Frontiers in Immunology*.

[42] Leyva-Castillo J. M., Yoon J., Geha R. S. (2019). IL-22 promotes allergic airway inflammation in epicutaneously sensitized mice. *Journal of Allergy and Clinical Immunology*.

[43] Ciofani M., Madar A., Galan C. (2012). A validated regulatory network for Th17 cell specification. *Cell*.

[44] Koh B., Hufford M. M., Pham D. (2016). The ETS family transcription factors Etv5 and PU.1 function in parallel to promote Th9 cell development. *The Journal of Immunology*.

[45] Campos Carrascosa L., Klein M., Kitagawa Y. (2017). Reciprocal regulation of the *Il9* locus by counteracting activities of transcription factors IRF1 and IRF4. *Nature Communications*.

[46] Goswami R., Jabeen R., Yagi R. (2012). STAT6-dependent regulation of Th9 development. *The Journal of Immunology*.

[47] Zhao P., Xiao X., Ghobrial R. M., Li X. C. (2013). IL-9 and Th9 cells: progress and challenges. *International Immunology*.

[48] Chang H. C., Sehra S., Goswami R. (2010). The transcription factor PU.1 is required for the development of IL-9-producing T cells and allergic inflammation. *Nature Immunology*.

[49] Gerlach K., Hwang Y., Nikolaev A. (2014). T_H_9 cells that express the transcription factor PU.1 drive T cell-mediated colitis via IL-9 receptor signaling in intestinal epithelial cells. *Nature Immunology*.

[50] Jabeen R., Goswami R., Awe O. (2013). Th9 cell development requires a BATF-regulated transcriptional network. *The Journal of Clinical Investigation*.

[51] Perumal N. B., Kaplan M. H. (2011). Regulating *Il9* transcription in T helper cells. *Trends in Immunology*.

[52] Kaplan M. H. (2017). The transcription factor network in Th9 cells. *Seminars in Immunopathology*.

[53] Liao W., Spolski R., Li P. (2014). Opposing actions of IL-2 and IL-21 on Th9 differentiation correlate with their differential regulation of BCL6 expression. *Proceedings of the National Academy of Sciences*.

[54] Malik S., Sadhu S., Elesela S. (2017). Transcription factor Foxo1 is essential for IL-9 induction in T helper cells. *Nature Communications*.

[55] Sakaguchi S., Sakaguchi N., Asano M., Itoh M., Toda M. (1995). Immunologic self-tolerance maintained by activated T cells expressing IL-2 receptor alpha-chains (CD25). Breakdown of a single mechanism of self-tolerance causes various autoimmune diseases. *The Journal of Immunology*.

[56] Nishizuka Y., Sakakura T. (1969). Thymus and reproduction: sex-linked dysgenesia of the gonad after neonatal thymectomy in mice. *Science*.

[57] Sakaguchi S., Yamaguchi T., Nomura T., Ono M. (2008). Regulatory T cells and immune tolerance. *Cell*.

[58] Roncarolo M. G., Gregori S., Bacchetta R., Battaglia M., Gagliani N. (2018). The biology of T regulatory type 1 cells and their therapeutic application in immune-mediated diseases. *Immunity*.

[59] Haribhai D., Williams J. B., Jia S. (2011). A requisite role for induced regulatory T cells in tolerance based on expanding antigen receptor diversity. *Immunity*.

[60] Ito T., Hanabuchi S., Wang Y. H. (2008). Two functional subsets of FOXP3^+^ regulatory T cells in human thymus and periphery. *Immunity*.

[61] Belkaid Y. (2007). Regulatory T cells and infection: a dangerous necessity. *Nature Reviews Immunology*.

[62] Shrestha S., Yang K., Guy C., Vogel P., Neale G., Chi H. (2015). T_reg_ cells require the phosphatase PTEN to restrain T_H_1 and T_FH_ cell responses. *Nature Immunology*.

[63] Akeus P., Langenes V., Kristensen J. (2015). Treg-cell depletion promotes chemokine production and accumulation of CXCR3(+) conventional T cells in intestinal tumors. *European Journal of Immunology*.

[64] Konkel J. E., Zhang D., Zanvit P. (2017). Transforming growth factor-*β* signaling in regulatory T cells controls T helper-17 cells and tissue-specific immune responses. *Immunity*.

[65] Wing K., Yamaguchi T., Sakaguchi S. (2011). Cell-autonomous and -non-autonomous roles of CTLA-4 in immune regulation. *Trends in Immunology*.

[66] Koizumi S. I., Sasaki D., Hsieh T. H. (2018). JunB regulates homeostasis and suppressive functions of effector regulatory T cells. *Nature Communications*.

[67] Ohue Y., Nishikawa H. (2019). Regulatory T (Treg) cells in cancer: Can Treg cells be a new therapeutic target?. *Cancer Science*.

[68] Deaglio S., Dwyer K. M., Gao W. (2007). Adenosine generation catalyzed by CD39 and CD73 expressed on regulatory T cells mediates immune suppression. *Journal of Experimental Medicine*.

[69] Burchill M. A., Yang J., Vogtenhuber C., Blazar B. R., Farrar M. A. (2006). IL-2 receptor *β*-dependent STAT5 activation is required for the development of Foxp3+regulatory T cells. *The Journal of Immunology*.

[70] Chen W., Jin W., Hardegen N. (2003). Conversion of peripheral CD4+CD25- naive T cells to CD4+CD25+ regulatory T cells by TGF-beta induction of transcription factor Foxp3. *Journal of Experimental Medicine*.

[71] Végran F., Berger H., Boidot R. (2014). The transcription factor IRF1 dictates the IL-21-dependent anticancer functions of T_H_9 cells. *Nature Immunology*.

[72] Vang K. B., Yang J., Mahmud S. A., Burchill M. A., Vegoe A. L., Farrar M. A. (2008). IL-2, -7, and -15, but not thymic stromal lymphopoeitin, redundantly govern CD4+Foxp3+ regulatory T cell development. *The Journal of Immunology*.

[73] Tai X., Cowan M., Feigenbaum L., Singer A. (2005). CD28 costimulation of developing thymocytes induces *Foxp3* expression and regulatory T cell differentiation independently of interleukin 2. *Nature Immunology*.

[74] Rudra D., deRoos P., Chaudhry A. (2012). Transcription factor Foxp3 and its protein partners form a complex regulatory network. *Nature Immunology*.

[75] Becher J., Simula L., Volpe E. (2018). AMBRA1 controls regulatory T-cell differentiation and homeostasis upstream of the FOXO3-FOXP3 axis. *Developmental Cell*.

[76] Feng Y., Arvey A., Chinen T., van der Veeken J., Gasteiger G., Rudensky A. Y. (2014). Control of the inheritance of regulatory T cell identity by a *cis* element in the *Foxp3* locus. *Cell*.

[77] Schlenner S. M., Weigmann B., Ruan Q. G., Chen Y., von Boehmer H. (2012). Smad3 binding to the foxp3 enhancer is dispensable for the development of regulatory T cells with the exception of the gut. *Journal of Experimental Medicine*.

[78] Kanamori M., Nakatsukasa H., Okada M., Lu Q., Yoshimura A. (2016). Induced regulatory T cells: their development, stability, and applications. *Trends in Immunology*.

[79] Wang Y. Q., Su M. A., Wan Y. S. Y. (2011). An essential role of the transcription factor GATA-3 for the function of regulatory T cells. *Immunity*.

[80] Ogawa C., Tone Y., Tsuda M., Peter C., Waldmann H., Tone M. (2013). TGF-beta-mediated Foxp3 gene expression is cooperatively regulated by Stat5, Creb, and AP-1 through CNS2. *Journal of Immunology*.

[81] Ghosh S., Roy-Chowdhuri S., Kang K., Im S. H., Rudra D. (2018). The transcription factor Foxp1 preserves integrity of an active Foxp3 locus in extrathymic Treg cells. *Nature Communications*.

[82] Vasanthakumar A., Moro K., Xin A. (2015). The transcriptional regulators IRF4, BATF and IL-33 orchestrate development and maintenance of adipose tissue-resident regulatory T cells. *Nature Immunology*.

[83] Seder R. A., Marth T., Sieve M. C. (1998). Factors involved in the differentiation of TGF-beta-producing cells from naive CD4+ T cells: IL-4 and IFN-gamma have opposing effects, while TGF-beta positively regulates its own production. *The Journal of Immunology*.

[84] Takami M., Love R. B., Iwashima M. (2012). TGF-*β* converts apoptotic stimuli into the signal for Th9 differentiation. *The Journal of immunology*.

[85] Anuradha R., George P. J., Hanna L. E. (2013). IL-4-, TGF-*β*–, and IL-1-dependent expansion of parasite antigen-specific Th9 cells is associated with clinical pathology in human lymphatic filariasis. *The Journal of Immunology*.

[86] Coombes J. L., Siddiqui K. R., Arancibia-Cárcamo C. V. (2007). A functionally specialized population of mucosal CD103+ DCs induces Foxp3+ regulatory T cells via a TGF-beta and retinoic acid-dependent mechanism. *Journal of Experimental Medicine*.

[87] Tone Y., Furuuchi K., Kojima Y., Tykocinski M. L., Greene M. I., Tone M. (2008). Smad3 and NFAT cooperate to induce *Foxp3* expression through its enhancer. *Nature Immunology*.

[88] Takimoto T., Wakabayashi Y., Sekiya T. (2010). Smad2 and Smad3 are redundantly essential for the TGF-*β*–mediated regulation of regulatory T plasticity and Th1 development. *The Journal of immunology*.

[89] Meylan F., Gomez-Rodriguez J. (2017). T cell receptor and co-stimulatory signals for Th9 generation. *Methods in Molecular Biology*.

[90] Jones C. P., Gregory L. G., Causton B., Campbell G. A., Lloyd C. M. (2012). Activin A and TGF-*β* promote T_H_9 cell-mediated pulmonary allergic pathology. *Journal of Allergy and Clinical Immunology*.

[91] Huber S., Stahl F. R., Schrader J. (2009). Activin a promotes the TGF-beta-induced conversion of CD4+CD25- T cells into Foxp3+ induced regulatory T cells. *The Journal of Immunology*.

[92] Bassil R., Orent W., Olah M. (2014). BCL6 controls Th9 cell development by repressing Il9 transcription. *The Journal of Immunology*.

[93] Glasmacher E., Agrawal S., Chang A. B. (2012). A genomic regulatory element that directs assembly and function of immune-specific AP-1-IRF complexes. *Science*.

[94] Alvisi G., Brummelman J., Puccio S. (2020). IRF4 instructs effector Treg differentiation and immune suppression in human cancer. *The Journal of Clinical Investigation*.

[95] Staudt V., Bothur E., Klein M. (2010). Interferon-regulatory factor 4 is essential for the developmental program of T helper 9 cells. *Immunity*.

[96] Lee W. H., Jang S. W., Kim H. S. (2019). BATF3 is sufficient for the induction of Il9 expression and can compensate for BATF during Th9 cell differentiation. *Experimental & Molecular Medicine*.

[97] Grinberg-Bleyer Y., Caron R., Seeley J. J. (2018). The alternative NF-*κ*B pathway in regulatory T cell homeostasis and suppressive function. *The Journal of Immunology*.

[98] Oh H., Ghosh S. (2013). NF-*κ*B: roles and regulation in different CD4+T-cell subsets. *Immunological Reviews*.

[99] Grinberg-Bleyer Y., Oh H., Desrichard A. (2017). NF-*κ*B c-Rel Is crucial for the regulatory T cell immune checkpoint in cancer. *Cell*.

[100] Oh H., Grinberg-Bleyer Y., Liao W. (2017). An NF-*κ*B transcription-factor-dependent lineage-specific transcriptional program promotes regulatory T cell identity and function. *Immunity*.

[101] Watts T. H. (2005). TNF/TNFR family members in costimulation of T cell responses. *Annual Review of Immunology*.

[102] Chi H. (2012). Regulation and function of mTOR signalling in T cell fate decisions. *Nature Reviews Immunology*.

[103] Bi E., Ma X., Lu Y. (2017). Foxo1 and Foxp1 play opposing roles in regulating the differentiation and antitumor activity of TH9 cells programmed by IL-7. *Science Signaling*.

[104] Wang Y., Bi Y., Chen X. (2016). Histone deacetylase SIRT1 negatively regulates the differentiation of interleukin-9-producing CD4^+^ T cells. *Immunity*.

[105] Chen H., Zhang L., Wang P. (2017). mTORC2 controls Th9 polarization and allergic airway inflammation. *Allergy*.

[106] Delgoffe G. M., Pollizzi K. N., Waickman A. T. (2011). The kinase mTOR regulates the differentiation of helper T cells through the selective activation of signaling by mTORC1 and mTORC2. *Nature Immunology*.

[107] Cai Z., Liu H., Wu X. (2017). Forkhead-box transcription factor 1 affects the apoptosis of natural regulatory T cells by controlling Aven expression. *BMC Immunology*.

[108] Chapman N. M., Zeng H., Nguyen T.-L. M. (2018). mTOR coordinates transcriptional programs and mitochondrial metabolism of activated T_reg_ subsets to protect tissue homeostasis. *Nature Communications*.

[109] Amsen D., Helbig C., Backer R. A. (2015). Notch in T cell differentiation: all things considered. *Trends in Immunology*.

[110] Bailis W., Yashiro-Ohtani Y., Fang T. C. (2013). Notch simultaneously orchestrates multiple helper T cell programs independently of cytokine signals. *Immunity*.

[111] Amsen D., Antov A., Jankovic D. (2007). Direct regulation of _Gata3_ expression determines the T helper differentiation potential of notch. *Immunity*.

[112] Doyle A. J., Redmond E. M., Gillespie D. L. (2015). Differential expression of Hedgehog/Notch and transforming growth factor-*β* in human abdominal aortic aneurysms. *Journal of Vascular Surgery*.

[113] Ting H. A., de Almeida Nagata D., Rasky A. J. (2018). Notch ligand delta-like 4 induces epigenetic regulation of Treg cell differentiation and function in viral infection. *Mucosal Immunology*.

[114] Lin C. L., Huang H. M., Hsieh C. L., Fan C. K., Lee Y. L. (2019). Jagged1-expressing adenovirus-infected dendritic cells induce expansion of Foxp3(+) regulatory T cells and alleviate T helper type 2-mediated allergic asthma in mice. *Immunology*.

[115] Huang M. T., Chen Y. L., Lien C. I. (2017). Notch ligand DLL4 alleviates allergic airway inflammation _via_ induction of a homeostatic regulatory pathway. *Scientific Reports*.

[116] Ferrandino F., Grazioli P., Bellavia D., Campese A. F., Screpanti I., Felli M. P. (2018). Notch and NF-*κ*B: coach and players of regulatory T-cell response in cancer. *Frontiers in Immunology*.

[117] Feuerer M., Hill J. A., Mathis D., Benoist C. (2009). Foxp3^+^ regulatory T cells: differentiation, specification, subphenotypes. *Nature Immunology*.

[118] Dominguez-Villar M., Hafler D. A. (2018). Regulatory T cells in autoimmune disease. *Nature Immunology*.

[119] Wilhelm C., Hirota K., Stieglitz B. (2011). An IL-9 fate reporter demonstrates the induction of an innate IL-9 response in lung inflammation. *Nature Immunology*.

[120] Tan C., Aziz M. K., Lovaas J. D. (2010). Antigen-specific Th9 cells exhibit uniqueness in their kinetics of cytokine production and short retention at the inflammatory site. *Journal of Immunology*.

[121] Hegazy A. N., Peine M., Helmstetter C. (2010). Interferons direct Th2 cell reprogramming to generate a stable GATA-3^+^T-bet^+^ cell subset with combined Th2 and Th1 cell functions. *Immunity*.

[122] Zhou X., Bailey-Bucktrout S. L., Jeker L. T. (2009). Instability of the transcription factor Foxp3 leads to the generation of pathogenic memory T cells *in vivo*. *Nature Immunology*.

[123] Kitz A., Dominguez-Villar M. (2017). Molecular mechanisms underlying Th1-like Treg generation and function. *Cellular and Molecular Life Sciences*.

[124] Koch M. A., Thomas K. R., Perdue N. R., Smigiel K. S., Srivastava S., Campbell D. J. (2012). T-bet^+^ Treg cells undergo abortive Th1 cell differentiation due to impaired expression of IL-12 receptor *β*2. *Immunity*.

[125] Levine A. G., Mendoza A., Hemmers S. (2017). Stability and function of regulatory T cells expressing the transcription factor T-bet. *Nature*.

[126] Noelle R. J., Nowak E. C. (2010). Cellular sources and immune functions of interleukin-9. *Nature Reviews Immunology*.

[127] Ulrich B. J., Verdan F. F., McKenzie A. N., Kaplan M. H., Olson M. R. (2017). STAT3 Activation Impairs the Stability of Th9 Cells. *The Journal of Immunology*.

[128] Shi X., Xiao X., Fan Y., Ghobrial R., Li X. (2015). GITR costimulation redirects regulatory T cells into Th9 cells to promote antitumor immunity. *Journal of Immunology*.

[129] Schlapbach C., Gehad A., Yang C. (2014). Human TH9 cells are skin-tropic and have autocrine and paracrine proinflammatory capacity. *Science Translational Medicine*.

[130] Jia L., Wang Y., Li J. (2017). Detection of IL-9 producing T cells in the PBMCs of allergic asthmatic patients. *BMC Immunology*.

[131] Yao W., Tepper R. S., Kaplan M. H. (2011). Predisposition to the development of IL-9-secreting T cells in atopic infants. *Journal of Allergy and Clinical Immunology*.

[132] Gomez-Rodriguez J., Meylan F., Handon R. (2016). Itk is required for Th9 differentiation via TCR-mediated induction of IL-2 and IRF4.

[133] Beale J., Jayaraman A., Jackson D. J. (2014). Rhinovirus-induced IL-25 in asthma exacerbation drives type 2 immunity and allergic pulmonary inflammation. *Science Translational Medicine*.

[134] Yao W., Zhang Y., Jabeen R. (2013). Interleukin-9 is required for allergic airway inflammation mediated by the cytokine TSLP. *Immunity*.

[135] Übel C., Sopel N., Graser A. (2014). The activating protein 1 transcription factor basic leucine zipper transcription factor, ATF-like (BATF), regulates lymphocyte- and mast cell- driven immune responses in the setting of allergic asthma. *Journal of Allergy and Clinical Immunology*.

[136] Mulrennan S. A., Redington A. E. (2004). Nitric oxide synthase inhibition: therapeutic potential in asthma. *Treatments in Respiratory Medicine*.

[137] Niedbala W., Besnard A. G., Nascimento D. C. (2014). Nitric oxide enhances Th9 cell differentiation and airway inflammation. *Nature Communications*.

[138] Zhao W., Tan C., Yu X., Yu R., Mei Q., Cheng Y. (2020). A 7-amino acid peptide mimic from hepatitis C virus hypervariable region 1 inhibits mouse lung Th9 cell differentiation by blocking CD81 signaling during allergic lung inflammation. *Journal of Immunology Research*.

[139] Palomares O., Yaman G., Azkur A. K., Akkoc T., Akdis M., Akdis C. A. (2010). Role of Treg in immune regulation of allergic diseases. *European Journal of Immunology*.

[140] Cohen R. I., Ye X., Ramdeo R., Liu S. F. (2019). The number and function of T regulatory cells in obese atopic female asthmatics. *Journal of Asthma*.

[141] Chen T., Hou X., Ni Y. (2018). The imbalance of FOXP3/GATA3 in regulatory T cells from the peripheral blood of asthmatic patients. *Journal of Immunology Research*.

[142] He Y.-T., Zhou Y., Shao Q. (2019). Immunoregulatory effects of subcutaneous immunotherapy on lymphocyte subgroups and cytokines in children with asthma. *Journal of Immunology Research*.

[143] Xu W., Lan Q., Chen M. (2012). Adoptive transfer of induced-Treg cells effectively attenuates murine airway allergic inflammation. *PLoS One*.

[144] Lu Y., Li Y., Zhou W., Ding B., Yu Q. (2019). Regulatory T cells regulate the distribution of natural killer T cells through CD39 signal transduction in asthma. *Human Cell*.

[145] Aron J. L., Akbari O. (2017). Regulatory T cells and type 2 innate lymphoid cell-dependent asthma. *Allergy*.

[146] Li M. O., Wan Y. Y., Sanjabi S., Robertson A. K. L., Flavell R. A. (2006). Transforming growth FACTOR-*β* regulation of immune responses. *Annual Review of Immunology*.

[147] Shamji M. H., Layhadi J. A., Achkova D. (2019). Role of IL-35 in sublingual allergen immunotherapy. *The Journal of Allergy and Clinical Immunology*.

[148] Akdis M., Verhagen J., Taylor A. (2004). Immune responses in healthy and allergic individuals are characterized by a fine balance between allergen-specific T regulatory 1 and T helper 2 cells. *Journal of Experimental Medicine*.

[149] Joetham A., Schedel M., O'Connor B. P. (2017). Inducible and naturally occurring regulatory T cells enhance lung allergic responses through divergent transcriptional pathways. *The Journal of Allergy and Clinical Immunology*.

[150] Charbonnier L. M., Cui Y., Stephen-Victor E. (2019). Functional reprogramming of regulatory T cells in the absence of Foxp3. *Nature Immunology*.

[151] Verbsky J. W., Chatila T. A. (2013). Immune dysregulation, polyendocrinopathy, enteropathy, X-linked (IPEX) and IPEX-related disorders: an evolving web of heritable autoimmune diseases. *Current Opinion in Pediatrics*.

[152] Josefowicz S. Z., Niec R. E., Kim H. Y. (2012). Extrathymically generated regulatory T cells control mucosal TH2 inflammation. *Nature*.

[153] Rivera Vargas T., Humblin E., Végran F., Ghiringhelli F., Apetoh L. (2017). TH9 cells in anti-tumor immunity. *Seminars in Immunopathology*.

[154] Miao B. P., Zhang R. S., Sun H. J. (2017). Inhibition of squamous cancer growth in a mouse model by staphylococcal enterotoxin B-triggered Th9 cell expansion. *Cellular & Molecular Immunology*.

[155] Zhao Y., Chu X., Chen J. (2016). Dectin-1-activated dendritic cells trigger potent antitumour immunity through the induction of Th9 cells. *Nature Communications*.

[156] Lu Y., Hong S., Li H. (2012). Th9 cells promote antitumor immune responses in vivo. *Journal of Clinical Investigation*.

[157] Park J., Li H., Zhang M. (2014). Murine Th9 cells promote the survival of myeloid dendritic cells in cancer immunotherapy. *Cancer Immunology Immunotherapy*.

[158] Rivera Vargas T., Cai Z., Shen Y. (2017). Selective degradation of PU.1 during autophagy represses the differentiation and antitumour activity of T_H_9 cells. *Nature Communications*.

[159] Xue G., Jin G., Fang J., Lu Y. (2019). IL-4 together with IL-1*β* induces antitumor Th9 cell differentiation in the absence of TGF-*β* signaling. *Nature Communications*.

[160] Mimoto F., Tatsumi K., Shimizu S. (2020). Exploitation of elevated extracellular ATP to specifically direct antibody to tumor microenvironment. *Cell Reports*.

[161] Roy S., Awasthi A. (2019). ATP triggers human Th9 cell differentiation via nitric oxide-mediated mTOR-HIF1*α* pathway. *Frontiers in Immunology*.

[162] Roychoudhuri R., Eil R. L., Restifo N. P. (2015). The interplay of effector and regulatory T cells in cancer. *Current Opinion in Immunology*.

[163] Luo C. T., Liao W., Dadi S., Toure A., Li M. O. (2016). Graded Foxo1 activity in T_reg_ cells differentiates tumour immunity from spontaneous autoimmunity. *Nature*.

[164] Plitas G., Konopacki C., Wu K. (2016). Regulatory T cells exhibit distinct features in human breast cancer. *Immunity*.

[165] de Simone M., Arrigoni A., Rossetti G. (2016). Transcriptional landscape of human tissue lymphocytes unveils uniqueness of tumor-infiltrating T regulatory cells. *Immunity*.

[166] Pardoll D. M. (2012). The blockade of immune checkpoints in cancer immunotherapy. *Nature Reviews Cancer*.

[167] Khaja A. S. S., Toor S. M., El Salhat H., Ali B. R., Elkord E. (2017). Intratumoral FoxP3(+)Helios(+) regulatory T cells upregulating immunosuppressive molecules are expanded in human colorectal cancer. *Frontiers in Immunology*.

[168] Sugiyama D., Nishikawa H., Maeda Y. (2013). Anti-CCR4 mAb selectively depletes effector-type FoxP3+CD4+ regulatory T cells, evoking antitumor immune responses in humans. *Proceedings of the National Academy of Sciences*.

[169] Chen M. L., Pittet M. J., Gorelik L. (2005). Regulatory T cells suppress tumor-specific CD8 T cell cytotoxicity through TGF-beta signals in vivo. *Proceedings of the National Academy of Sciences of the United States of America*.

[170] Hao S., Chen X., Wang F. (2018). Breast cancer cell-derived IL-35 promotes tumor progression via induction of IL-35-producing induced regulatory T cells. *Carcinogenesis*.

[171] Jang J. E., Hajdu C. H., Liot C., Miller G., Dustin M. L., Bar-Sagi D. (2017). Crosstalk between regulatory T cells and tumor-associated dendritic cells negates anti-tumor immunity in pancreatic cancer. *Cell Reports*.

[172] Quezada S. A., Peggs K. S., Curran M. A., Allison J. P. (2006). CTLA4 blockade and GM-CSF combination immunotherapy alters the intratumor balance of effector and regulatory T cells. *Journal of Clinical Investigation*.

[173] Bos P. D., Plitas G., Rudra D., Lee S. Y., Rudensky A. Y. (2013). Transient regulatory T cell ablation deters oncogene-driven breast cancer and enhances radiotherapy. *Journal of Experimental Medicine*.

[174] Teng M. W., Ngiow S. F., von Scheidt B., McLaughlin N., Sparwasser T., Smyth M. J. (2010). Conditional regulatory T-cell depletion releases adaptive immunity preventing carcinogenesis and suppressing established tumor growth. *Cancer Research*.

[175] Liu X., Mo W., Ye J. (2018). Regulatory T cells trigger effector T cell DNA damage and senescence caused by metabolic competition. *Nature Communications*.

[176] Campbell D. J., Koch M. A. (2011). Phenotypical and functional specialization of FOXP3^+^ regulatory T cells. *Nature Reviews Immunology*.

[177] Duhen T., Duhen R., Lanzavecchia A., Sallusto F., Campbell D. J. (2012). Functionally distinct subsets of human FOXP3+ Treg cells that phenotypically mirror effector Th cells. *Blood*.

[178] Salazar Y., Zheng X., Brunn D. (2020). Microenvironmental Th9 and Th17 lymphocytes induce metastatic spreading in lung cancer. *Journal of Clinical Investigation*.

[179] Nishikawa H., Sakaguchi S. (2010). Regulatory T cells in tumor immunity. *International Journal of Cancer*.

[180] Klages K., Mayer C. T., Lahl K. (2010). Selective depletion of Foxp3+ regulatory T cells improves effective therapeutic vaccination against established melanoma. *Cancer Res*.

[181] Cui M., Lv Y., Lu J. (2018). Decreased frequency of circulating Th9 cells in patients with chronic hepatitis B infection. *Journal of Clinical Laboratory Analysis*.

[182] Ouyang W., Beckett O., Flavell R. A., Li M. O. (2009). An essential role of the forkhead-box transcription factor Foxo1 in control of T cell homeostasis and tolerance. *Immunity*.

[183] Lin L., Hron J. D., Peng S. L. (2004). Regulation of NF-*κ*B, Th activation, and autoinflammation by the forkhead transcription factor Foxo3a. *Immunity*.

[184] Lucca L. E., Dominguez-Villar M. (2020). Modulation of regulatory T cell function and stability by co-inhibitory receptors. *Nature Reviews Immunology*.

[185] Delgoffe G. M., Kole T. P., Zheng Y. (2009). The mTOR kinase differentially regulates effector and regulatory T cell lineage commitment. *Immunity*.

[186] Kerdiles Y. M., Stone E. L., Beisner D. R. (2010). Foxo transcription factors control regulatory T cell development and function. *Immunity*.

[187] Ouyang W., Beckett O., Ma Q., Paik J. H., DePinho R. A., Li M. O. (2010). Foxo proteins cooperatively control the differentiation of Foxp3^+^ regulatory T cells. *Nature Immunology*.

[188] Fu Y., Wang J., Panangipalli G. (2020). STAT5 promotes accessibility and is required for BATF-mediated plasticity at the *Il9* locus. *Nature Communications*.

[189] Nguyen K. D., Vanichsarn C., Nadeau K. C. (2010). TSLP directly impairs pulmonary Treg function: association with aberrant tolerogenic immunity in asthmatic airway. *Allergy, Asthma & Clinical Immunology*.

[190] Ogasawara T., Kohashi Y., Ikari J. (2018). Allergic TH2 response governed by B-cell lymphoma 6 function in naturally occurring memory phenotype CD4(+) T cells. *Frontiers in Immunology*.

[191] Sawant D. V., Wu H., Yao W., Sehra S., Kaplan M. H., Dent A. L. (2015). The transcriptional repressor Bcl6 controls the stability of regulatory T cells by intrinsic and extrinsic pathways. *Immunology*.

[192] Sawant D. V., Sehra S., Nguyen E. T. (2012). Bcl6 controls the Th2 inflammatory activity of regulatory T cells by repressing Gata3 function. *Journal of Immunology*.

[193] Li Y., Wang Z., Lin H. (2020). Bcl6 preserves the suppressive function of regulatory T cells during tumorigenesis. *Frontiers in Immunology*.

[194] Xiao X., Gong W., Demirci G. (2012). New insights on OX40 in the control of T cell immunity and immune tolerance in vivo. *Journal of Immunology*.

[195] Xiao X., Fan Y., Li J. (2018). Guidance of super-enhancers in regulation of IL-9 induction and airway inflammation. *Journal of Experimental Medicine*.

[196] Zhang X. L., Xiao X., Lan P. X. (2018). OX40 costimulation inhibits Foxp3 expression and Treg induction via BATF3-dependent and independent mechanisms. *Cell Reports*.

[197] Hirschhorn-Cymerman D., Rizzuto G. A., Merghoub T. (2009). OX40 engagement and chemotherapy combination provides potent antitumor immunity with concomitant regulatory T cell apoptosis. *Journal of Experimental Medicine*.

[198] Paulsen M., Valentin S., Mathew B. (2011). Modulation of CD4^+^ T-cell activation by CD95 co- stimulation. *Cell Death & Differentiation*.

[199] Meyer zu Horste G., Przybylski D., Schramm M. A. (2018). Fas promotes T helper 17 cell differentiation and inhibits T helper 1 cell development by binding and sequestering transcription factor STAT1. *Immunity*.

[200] Shen Y., Song Z., Lu X. (2019). Fas signaling-mediated T_H_9 cell differentiation favors bowel inflammation and antitumor functions. *Nature Communications*.

[201] Weiss J. M., Subleski J. J., Back T. (2014). Regulatory T cells and myeloid-derived suppressor cells in the tumor microenvironment undergo Fas-dependent cell death during IL-2/*α*CD40 therapy. *Journal of Immunology*.

[202] Zeng H., Chi H. (2015). Metabolic control of regulatory T cell development and function. *Trends in Immunology*.

[203] Waickman A. T., Powell J. D. (2012). mTOR, metabolism, and the regulation of T-cell differentiation and function. *Immunological Reviews*.

[204] Yang H., Bi Y., Xue L. (2015). Multifaceted modulation of SIRT1 in cancer and inflammation. *Critical Reviews in Oncogenesis*.

[205] Kwon H. S., Lim H. W., Wu J., Schnölzer M., Verdin E., Ott M. (2012). Three novel acetylation sites in the Foxp3 transcription factor regulate the suppressive activity of regulatory T cells. *Journal of Immunology*.

[206] Marcel N., Perumalsamy L. R., Shukla S. K., Sarin A. (2017). The lysine deacetylase sirtuin 1 modulates the localization and function of the Notch1 receptor in regulatory T cells. *Science Signaling*.

[207] Søndergaard K. L., Hilton D. A., Penney M., Ollerenshaw M., Demaine A. G. (2002). Expression of hypoxia-inducible factor 1alpha in tumours of patients with glioblastoma. *Neuropathology and Applied Neurobiology*.

[208] Miska J., Lee-Chang C., Rashidi A. (2019). HIF-1*α* is a metabolic switch between glycolytic-driven migration and oxidative phosphorylation-driven immunosuppression of Tregs in glioblastoma. *Cell Reports*.

[209] Huang Y. Y., Jiang H. X., Shi Q. Y. (2020). miR-145 inhibits Th9 cell differentiation by suppressing activation of the PI3K/Akt/mTOR/p70S6K/HIF-1*α* pathway in malignant ascites from liver cancer. *OncoTargets and Therapy*.

[210] Tan H., Wang S., Zhao L. (2017). A tumour-promoting role of Th9 cells in hepatocellular carcinoma through CCL20 and STAT3 pathways. *Clinical and Experimental Pharmacology and Physiology*.

[211] Gounaris E., Blatner N. R., Dennis K. (2009). T-regulatory cells shift from a protective anti-inflammatory to a cancer-promoting proinflammatory phenotype in polyposis. *Cancer Research*.

[212] di Pilato M., Kim E. Y., Cadilha B. L. (2019). Targeting the CBM complex causes T_reg_ cells to prime tumours for immune checkpoint therapy. *Nature*.

[213] Massoud A. H., Charbonnier L. M., Lopez D., Pellegrini M., Phipatanakul W., Chatila T. A. (2016). An asthma-associated _IL4R_ variant exacerbates airway inflammation by promoting conversion of regulatory T cells to T_H_17-like cells. *Nature Medicine*.

[214] Hsu T. S., Lin Y. L., Wang Y. A. (2020). HIF-2*α* is indispensable for regulatory T cell function. *Nature Communications*.

[215] Mayer A., Schneider F., Vaupel P., Sommer C., Schmidberger H. (2012). Differential expression of HIF-1 in glioblastoma multiforme and anaplastic astrocytoma. *International Journal of Oncology*.

[216] Sitkovsky M. V. (2009). T regulatory cells: hypoxia-adenosinergic suppression and re-direction of the immune response. *Trends in Immunology*.

[217] Krishnamoorthy N., Khare A., Oriss T. B. (2012). Early infection with respiratory syncytial virus impairs regulatory T cell function and increases susceptibility to allergic asthma. *Nature Medicine*.

[218] Xin L., Gao J., Ge X. (2018). Increased pro-inflammatory cytokine-secreting regulatory T cells are correlated with the plasticity of T helper cell differentiation and reflect disease status in asthma. *Respiratory Medicine*.

